# Hippocampal transcriptome-wide association study and neurobiological pathway analysis for Alzheimer’s disease

**DOI:** 10.1371/journal.pgen.1009363

**Published:** 2021-02-25

**Authors:** Nana Liu, Jiayuan Xu, Huaigui Liu, Shijie Zhang, Miaoxin Li, Yao Zhou, Wen Qin, Mulin Jun Li, Chunshui Yu, for the Alzheimer’s disease Neuroimaging Initiative

**Affiliations:** 1 Department of Radiology and Tianjin Key Laboratory of Functional Imaging, Tianjin Medical University General Hospital, Tianjin, China; 2 The Province and Ministry Co-sponsored Collaborative Innovation Center for Medical Epigenetics, Tianjin Key Laboratory of Medical Epigenetics, Department of Pharmacology, Tianjin Medical University, Tianjin, China; 3 Department of Medical Genetics, Center for Genome Research, Zhongshan School of Medicine, Sun Yat-sen University, Guangzhou, China; 4 Centre for Genomic Sciences, The University of Hong Kong, Hong Kong Special Administrative Region, China; 5 Department of Psychiatry, The University of Hong Kong, Hong Kong Special Administrative Region, China; 6 Centre for Reproduction, Development and Growth, Li Ka Shing Faculty of Medicine, The University of Hong Kong, Hong Kong Special Administrative Region, China; 7 Chinese Academy of Sciences (CAS) Center for Excellence in Brain Science and Intelligence Technology, Chinese Academy of Sciences, Shanghai, China; Loyola University Chicago, UNITED STATES

## Abstract

Genome-wide association studies (GWASs) have identified multiple susceptibility loci for Alzheimer’s disease (AD), which is characterized by early and progressive damage to the hippocampus. However, the association of hippocampal gene expression with AD and the underlying neurobiological pathways remain largely unknown. Based on the genomic and transcriptomic data of 111 hippocampal samples and the summary data of two large-scale meta-analyses of GWASs, a transcriptome-wide association study (TWAS) was performed to identify genes with significant associations between hippocampal expression and AD. We identified 54 significantly associated genes using an AD-GWAS meta-analysis of 455,258 individuals; 36 of the genes were confirmed in another AD-GWAS meta-analysis of 63,926 individuals. Fine-mapping models further prioritized 24 AD-related genes whose effects on AD were mediated by hippocampal expression, including *APOE* and two novel genes (*PTPN9* and *PCDHA4*). These genes are functionally related to amyloid-beta formation, phosphorylation/dephosphorylation, neuronal apoptosis, neurogenesis and telomerase-related processes. By integrating the predicted hippocampal expression and neuroimaging data, we found that the hippocampal expression of *QPCTL* and *ERCC2* showed significant difference between AD patients and cognitively normal elderly individuals as well as correlated with hippocampal volume. Mediation analysis further demonstrated that hippocampal volume mediated the effect of hippocampal gene expression (*QPCTL* and *ERCC2*) on AD. This study identifies two novel genes associated with AD by integrating hippocampal gene expression and genome-wide association data and reveals candidate hippocampus-mediated neurobiological pathways from gene expression to AD.

## Author summary

The hippocampus is a potential neuroimaging endophenotype for Alzheimer’s disease (AD). This study identifies genes whose expression in hippocampal tissue is associated with AD and establishes the pathways from hippocampal gene expression to hippocampal volume to AD. We demonstrate that 24 genes are associated with AD in hippocampal tissue, and these genes are enriched for AD-related biological processes of amyloid-beta formation, phosphorylation/dephosphorylation, neuronal apoptosis, neurogenesis and telomerase-related processes. We further show that hippocampal volume mediates the effects of the hippocampal gene expression of *QPCTL* and *ERCC2* on AD. These findings improve our understanding of the genetic and neural mechanisms of AD.

## Introduction

Alzheimer’s disease (AD) is a neurodegenerative disorder clinically characterized by progressive dementia and pathologically featured by senile plaques composed of amyloid beta peptide (Aβ) and intracellular neurofibrillary tangles (NFTs), which themselves are composed of hyperphosphorylated tau [1,2]. AD is a highly heritable disease with an estimated heritability of 58%-79% [3], emphasizing the importance of exploring the genetic mechanisms of AD. Despite rapid progress in utilizing genome-wide association studies (GWASs) and meta-analyses to identify AD-related genetic variants [4–11], the pathogenic mechanisms of the identified genetic loci in AD remain largely unknown.

Expression quantitative trait loci (eQTLs) are considered links between GWAS loci and disease susceptibility [12,13]. By integrating the large-scale gene expression data of a given tissue and disease-related GWAS data, transcriptome-wide association study (TWAS) has been proposed as a powerful approach to identify genes with significant associations between gene expression in certain tissues and the disease of interest [14–16]. By incorporating transcriptomic data of available human tissues and GWAS data of AD, several TWAS studies have confirmed multiple AD-related genes identified by GWASs and found novel genes that have not been previously reported [17–21]. Although the inclusion of all available tissues in these TWAS studies could improve the power, they provide little tissue-specific information, which is important for exploring pathogenic mechanisms of AD because tissues show different eQTLs [22] and TWAS is more reliable for trait-related tissues than for trait-unrelated tissues [23].

In neuroimaging studies, hippocampal atrophy is the most prominent imaging feature of AD [24–26]. Most of the neuropathological hallmarks (neurofibrillary tangles, neuronal loss, synaptic loss, amyloid plaques, and glial responses) of AD can be observed in the hippocampus, and neurofibrillary tangles, neuronal and synaptic loss are present in the hippocampus at an early stage of AD, which are closely associated with the progression of AD [27]. Moreover, eQTLs of hippocampal tissue are significantly enriched for AD-GWAS-identified associations [28]. These findings indicate that hippocampal tissue is an ideal candidate for AD-TWAS and that hippocampal volume is a potential neuroimaging marker to investigate the mediation effect of the hippocampus on the association between hippocampal gene expression and AD.

**Fig 1:**
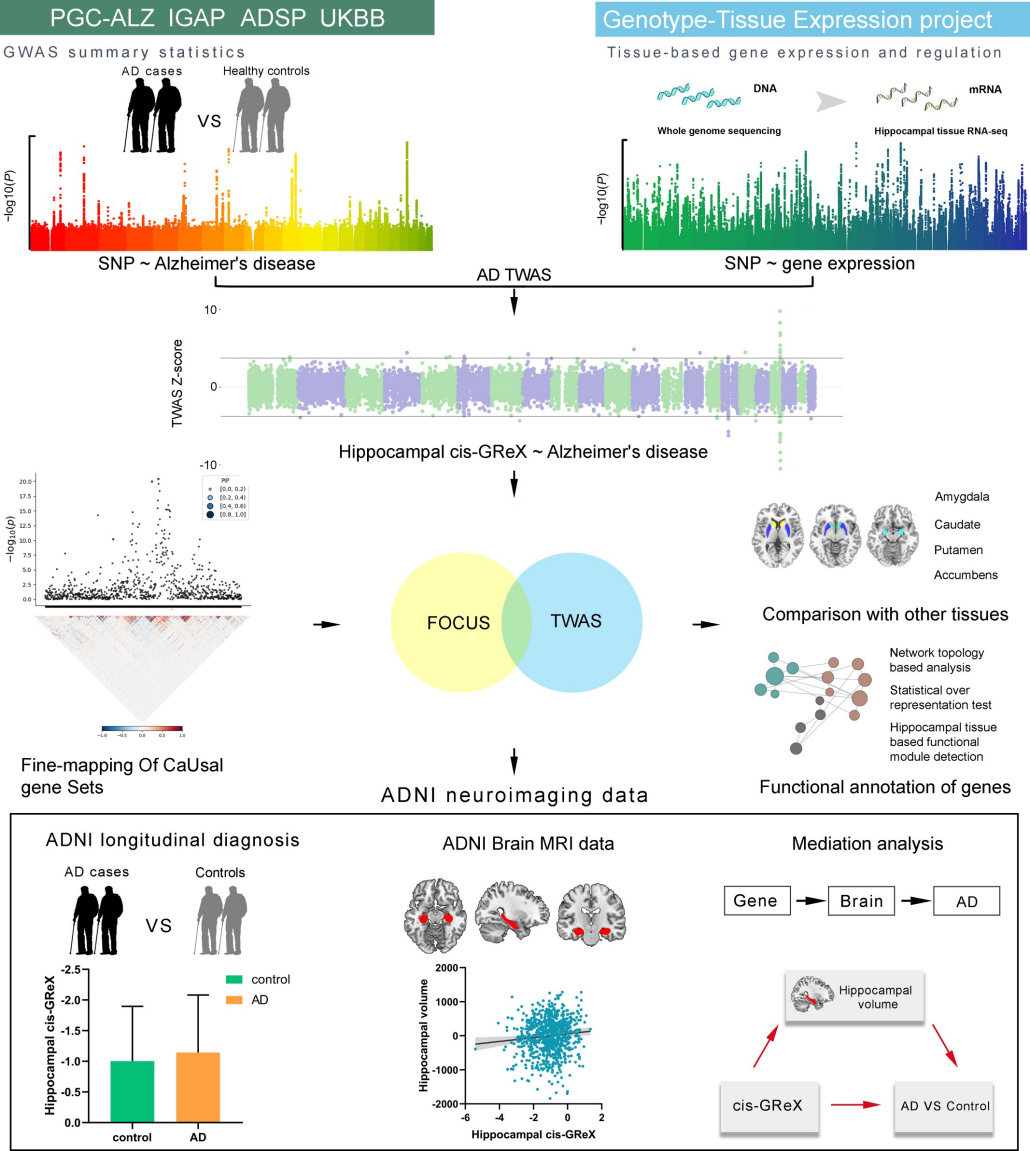
A schematic overview of the study design. AD = Alzheimer’s disease; ADNI = Alzheimer’s disease Neuroimaging Initiative; ADSP = Alzheimer’s disease Sequencing Project; *cis*-GReX = *cis*-genetically regulated expression; FOCUS = Fine-mapping of causal gene sets; GWAS = Genome-wide association study; IGAP = International Genomics of Alzheimer’s Project; PGC-ALZ = Alzheimer’s disease working group of the Psychiatric Genomics Consortium; TWAS = Transcriptome-wide association study; UKBB = UK Biobank.

In this study, we first determined the relationship between each single nucleotide polymorphism (SNP) and hippocampal gene expression using whole genome sequencing (WGS) data and hippocampal tissue RNA-seq data provided by GTEx [22]. Second, based on the obtained SNP-expression associations and SNP-AD associations identified by AD-GWAS, TWAS was performed to identify hippocampus- and AD-related genes, which were defined as genes whose *cis*-genetically regulated expression (*cis*-GReX) in hippocampal tissue was associated with AD. Third, fine-mapping analysis was used to prioritize these genes, and associations of the expression of these genes in four other subcortical tissues (amygdala, caudate, nucleus accumbens and putamen) with AD were also studied. The identified genes were functionally annotated by network topology-based analysis, statistical over-representation test and hippocampal-based functional module detection. Finally, we further validated the identified genes in Alzheimer’s Disease Neuroimaging Initiative (ADNI) neuroimaging data and established the pathway from hippocampal gene expression to hippocampal volume to AD diagnosis. A schematic overview of the study design is presented in Fig 1.

## Results

### Prediction models for hippocampal gene expression

**Fig 2:**
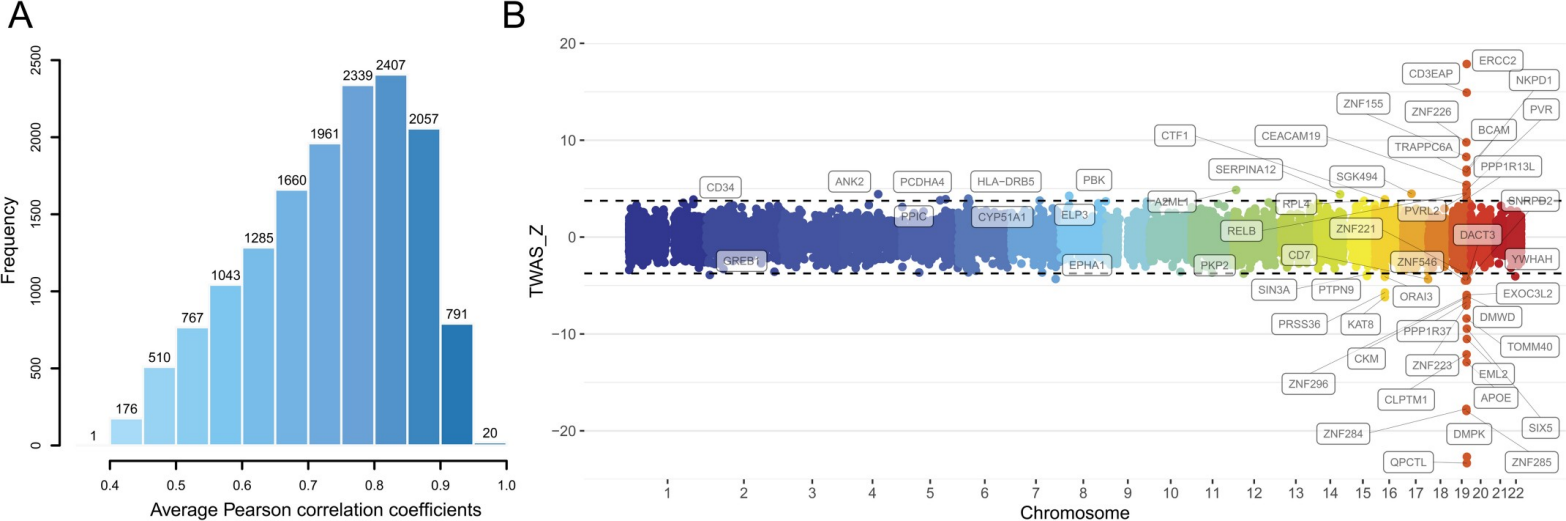
**Gene expression prediction models of hippocampal tissue (A) and TWAS results of AD (B)**. (A) Average Pearson correlation coefficients of 15,017 significant hippocampal gene expression prediction models (*p*_*c*_ < 0.05, FWE corrected). (B) Manhattan plot of all TWAS associations of AD in the discovery stage. Each point represents a single gene, with physical position in chromosome plotted on the x-axis and z-score of the association statistics between gene and AD plotted on the y-axis. Transcriptome-wide significant threshold (*q*_*c*_ < 0.05, FDR corrected) is highlighted as black dotted lines and the significant associations are labeled with gene names. AD, Alzheimer’s disease; FDR, false discovery rate; FWE, familywise error; TWAS, transcriptome-wide association study.

We established correspondences between SNPs and the expression of each gene in hippocampal tissue and calculated the weighted value of each SNP in predicting the expression of the gene using the WGS and RNA-seq data of 111 hippocampal samples from GTEx. For each gene, QTLtools (https://qtltools.github.io/qtltools/) [29] was used to perform conditional analysis to identify *cis*-eQTLs with independent effects on gene expression. The GTEx V7 pipeline (https://github.com/hakyimlab/PredictDB_Pipeline_GTEx_v7) was applied to train prediction models for hippocampal expression of 15,831 protein-coding genes with the nested cross validated elastic-net [14]. A prediction model was significant if the average Pearson correlation coefficient between predicted and measured gene expression during nested cross validation was greater than 0.1 and the estimated *p*-value for this statistic passed the multiple testing correction threshold of familywise error (FWE) (*p*_*c*_ < 0.05/15,831 = 3.16 × 10^−6^). Among the 15,831 protein-coding genes, we built significant hippocampal gene expression prediction models for 15,017 genes (*p*_*c*_ < 0.05, FWE corrected), indicating a rather high success rate (94.9%). The average Pearson correlation coefficients between predicted and observed expression levels during nested cross validation in hippocampal tissue for these genes (mean ± SD = 0.73 ± 0.12, range 0.40–0.96) are shown in Fig 2A, which were relatively high, suggesting good performance of these prediction models. For each gene, the prediction model generated the weighted value for each SNP’s relative contribution to the gene’s expression level in hippocampal tissue.

### Identification and validation of AD-related genes using TWAS

TWAS can integrate the gene expression of certain tissues and GWAS data to test correlations between *cis*-GReX and disease/complex traits [14–16]. In our study, summary-PrediXcan (S-PrediXcan) [15] was used to perform TWAS to identify significant associations between AD and gene expression in hippocampal tissue. Two sets of GWAS summary statistics of AD were used: the meta-analysis (n = 455,258 including 71,880 AD or AD-by-proxy and 383,378 controls) [4] collected from the Alzheimer’s disease working group of the Psychiatric Genomics Consortium (PGC-ALZ), the International Genomics of Alzheimer’s Project (IGAP), the Alzheimer’s Disease Sequencing Project (ADSP) and the UK Biobank (UKBB) was used as the discovery sample. The updated GWAS meta-analysis of IGAP (n = 63,926 including 21,982 AD and 41,944 controls) [11] was used as the replication sample. Based on SNP-AD associations obtained from the GWAS summary statistics of 455,258 individuals (the discovery sample) and SNP-expression associations obtained from the hippocampal gene expression prediction models, we found 54 genes whose *cis*-GReX values in hippocampal tissue were significantly associated with AD at the 5% false discovery rate (FDR) threshold (*q*_*c*_ < 0.05) (Fig 2B and S1 Table). Based on the GWAS summary statistics of 63,926 individuals from IGAP (the replication sample) and the hippocampal gene expression prediction models, we successfully replicated 36 of 54 genes at a nominal threshold of *p* < 0.05 with consistent direction of z-scores between discovery and validation stage, among which 23 genes passed the FDR correction for multiple testing (*q*_*c*_ < 0.05) in the replication samples (S1 Fig and S1 Table).

### Fine-mapping prioritizes AD-related genes

**Fig 3:**
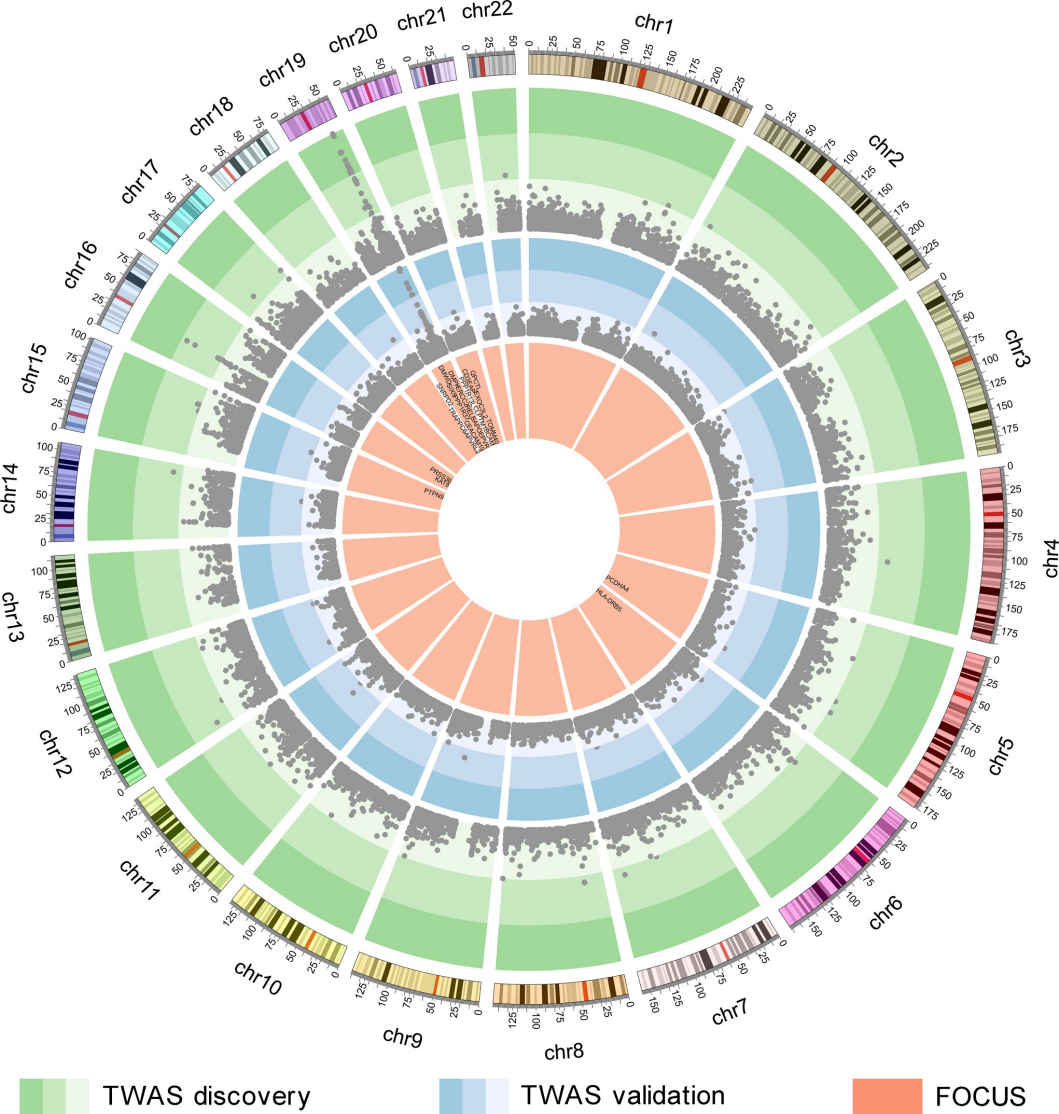
Genes identified by TWAS and FOCUS. The green circle shows the genes identified by the AD-TWAS in hippocampal tissue, each point represents a single gene, with physical position in human genome plotted on the x-axis and -log_10_(*p*) of association between *cis*-GReX in hippocampal tissue and AD plotted on the y-axis. The color gradients represent significant levels and points located in the green and darker green regions indicate significant associations with AD at the 5% FDR threshold. The blue circle shows the AD-TWAS results of validation stage. The orange circle shows the results of FOCUS, the 24 genes showed in the figure are included in 90% credible gene sets. AD, Alzheimer’s disease; *cis*-GReX, *cis*-genetically regulated expression; FDR, false discovery rate; FOCUS, fine-mapping of causal gene sets; TWAS, transcriptome-wide association study.

The reliability of TWAS-identified AD-related genes was challenged by linkage disequilibrium (LD) among the SNPs and coregulation of gene models [23]. Here, FOCUS (fine-mapping of causal gene sets) [30] was further used to prioritize the 36 genes for AD by assigning a probability for each gene based on prediction modules, recommended LD reference data, and AD-GWAS summary statistics (n = 455,258 including 71,880 AD or AD-by-proxy and 383,378 controls). FOCUS inferred whether each of the 36 TWAS-identified genes was included in credible set at the nominal confidence level (90%). Among the 36 TWAS-identified AD-related genes, FOCUS prioritized 24, which were in credible sets (Fig 3 and S1 Table).

### Specificity for hippocampal tissue

**Fig 4:**
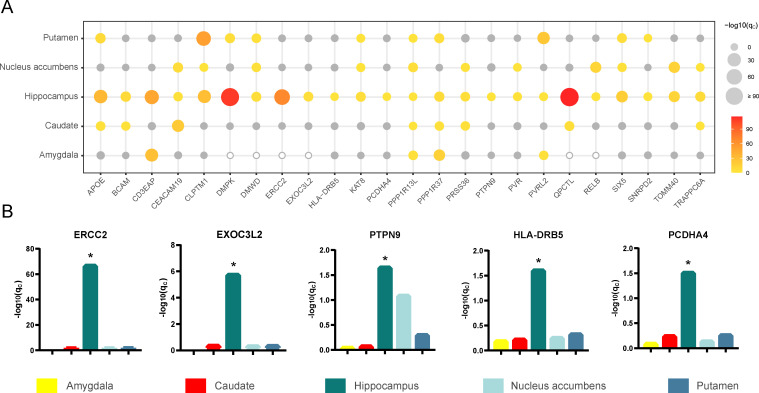
TWAS results of different subcortical tissues. (A) The bubble plot shows TWAS results of different subcortical tissues. The x-axis shows the 24 prioritized AD-related genes, the y-axis shows the five types of brain tissues. The size and the color of the bubbles demonstrate the significance of each gene in TWAS of a given tissue. The gray bubbles represent non-significant associations in TWAS, the hollow bubbles reflect the genes whose prediction models are not established successfully. (B) The bar plots show the FDR corrected *p*-values in TWAS for the five genes associated with AD only in hippocampal tissue (*q*_*c*_ < 0.05, FDR corrected). AD, Alzheimer’s disease; FDR, false discovery rate; TWAS, transcriptome-wide association study. *q_*c*_ < 0.05.

To determine whether the 24 identified AD-related genes specifically affect the hippocampus, we also investigated the associations of the expression of these genes in four other subcortical tissues with AD. These subcortical tissues included the amygdala, caudate, nucleus accumbens and putamen, and the volume loss of the latter two nuclei appears earlier than that of the hippocampus in AD patients [31]. The same pipeline used for the hippocampus was applied to train prediction models and to perform TWAS for the other four tissues. The average Pearson correlation coefficients between predicted and observed expression levels for the amygdala, caudate, nucleus accumbens and putamen are shown in S2 Fig. We used the prediction models of the four tissues and the AD-GWAS summary statistics (n = 455,258 including 71,880 AD or AD-by-proxy and 383,378 controls) to perform TWAS. Manhattan plots of the TWAS results are shown in S3–S6 Figs. We compared the TWAS results of the 24 prioritized AD-related genes between hippocampal tissue and other tissues (Fig 4A). The gene expression prediction models of six genes were not established successfully in the amygdala, which meant that the SNPs could not predict the gene expression in this tissue. The gene expression of *ERCC2*, *EXOC3L2*, *PTPN9*, *HLA-DRB5* and *PCDHA4* was associated with AD only in hippocampal tissue at the 5% FDR threshold (Fig 4B). More genes showed shared genetic contributions to AD in at least two tissues. For example, AD was affected by the gene expression of *CD3EAP* in the hippocampus and amygdala; *TOMM40*, *PVR* and *RELB* in the hippocampus and nucleus accumbens; *DMPK* and *SNRPD2* in the hippocampus and putamen; and *QPCTL* and *BCAM* in the hippocampus and caudate. In addition, some genes showed extensive cross-tissue effects on AD, such as AD was associated with the expression of *APOE*, *CEACAM19*, *CLPTM1*, *DMWD*, *KAT8*, *PRSS36*, *PVRL2*, *SIX5*, *TRAPPC6A*, *PPP1R13L* and *PPP1R37* in at least three tissues.

### Functional annotation of AD-related genes

**Fig 5:**
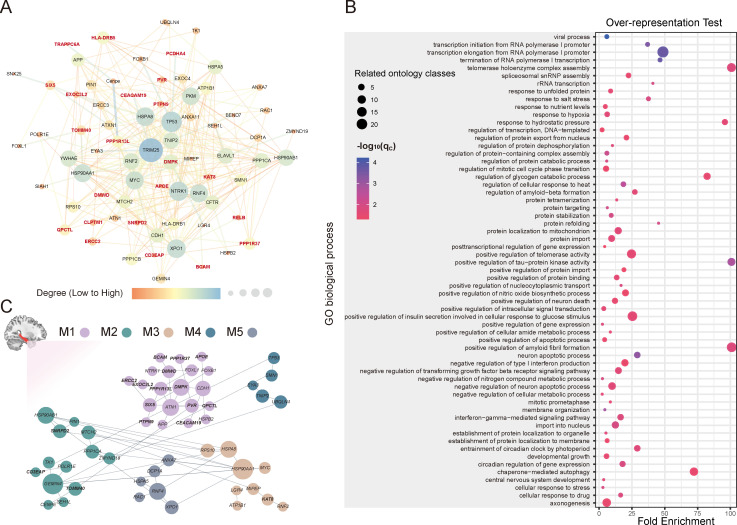
Functional annotation of AD-related genes. (A) The PPI network containing 22 seed proteins (marked in bold and dark red) and 50 top-ranking neighbors. (B) The bubble plot shows the enriched GO terms of biological process (the most specific subclass of each ontology is shown). The x-axis shows the fold enrichment of statistical over-representation test for each term (y-axis). The size of the bubbles reflects the number of related enriched terms of biological process. The color of the bubbles demonstrates the significance of each term based on the statistical over-representation test. (C) Hippocampal-based functional modules formed by AD-TWAS genes (marked in bold) and tightly connected genes. AD, Alzheimer’s disease; FDR, false discovery rate; GO, gene ontology; M, module; PPI, protein-protein interaction; TWAS, transcriptome-wide association study.

To identify the functional relationship and the involved biological processes of the 24 prioritized AD-related genes, we first constructed a protein-protein interaction (PPI) network by network topology-based analysis embedded in the WEB-based gene set analysis toolkit (Webgestalt, https://www.webgestalt.org) [32]. The PPI network contained 22 seed genes (prioritized AD-related genes) and 50 top-ranking neighbors based on network proximity (Fig 5A and S2 Table), *PVRL2* and *PRSS36* were not included in the network due to the lack of connectivity. APP, a known susceptibility protein of AD [33], was a hub node of the network. Notably, the prioritized gene *QPCTL* was directly connected with *APP*, suggesting their molecular interaction and AD relevance. In addition, the AD-related genes we identified were included in the common network with *APP*, which means that they may have coherent biological functions. Second, the statistical over-representation test of PANTHER [34] was used to identify enriched gene ontology (GO) terms of biological process for the generated network. The 72 genes in the network were enriched in 260 GO biological process terms (*q*_*c*_ < 0.05, Benjamini-Hochberg FDR (BH-FDR) corrected) (S3 Table). These GO terms were divided into different ontologies according to hierarchical relations. Specifically, the 72 genes were mainly over-represented in a GO subclass of biological processes for neuron-related functions, such as neuron apoptotic process (fold enrichment = 28.56, *q*_*c*_ = 0.000776), positive regulation of neuron death (fold enrichment = 11.54, *q*_*c*_ = 0.0324), negative regulation of neuron apoptotic process (fold enrichment = 9.27, *q*_*c*_ = 0.0215), axonogenesis (fold enrichment = 5.07, *q*_*c*_ = 0.0344), and central nervous system development (fold enrichment = 3.34, *q*_*c*_ = 0.0215). The genes were also correlated with amyloid-beta and tau phosphorylation related processes, including positive regulation of amyloid fibril formation (fold enrichment > 100, *q*_*c*_ = 0.0181), regulation of amyloid-beta formation (fold enrichment = 26.78, *q*_*c*_ = 0.022), positive regulation of tau-protein kinase activity (fold enrichment > 100, *q*_*c*_ = 0.00178), and regulation of protein dephosphorylation (fold enrichment = 9.99, *q*_*c*_ = 0.0174), which are well-known neuropathology of AD. In addition, they were associated with telomerase-related processes, such as telomerase holoenzyme complex assembly (fold enrichment > 100, *q*_*c*_ = 0.018) and positive regulation of telomerase activity (fold enrichment = 23.8, *q*_*c*_ = 0.0267) (Fig 5B). These results demonstrated that the 22 prioritized AD-related genes were interconnected in a common PPI network and contributed to the neuropathological process of AD.

### Modulization analysis of AD-related genes in the hippocampal network

Functional modules were built using the HumanBase online tool [35] (https://hb.flatironinstitute.org/) in the context of hippocampal tissue networks. The 72 genes in the constructed PPI network were clustered into five cohesive functional modules in hippocampal tissue. Module 1 (M1) included 13/24 prioritized AD-related genes (*APOE*, *BCAM*, *CEACAM19*, *DMPK*, *DMWD*, *ERCC2*, *EXOC3L2*, *PPP1R13L*, *PPP1R37*, *PTPN9*, *PVR*, *QPCTL* and *SIX5*), module 2 (M2) contained 3/24 prioritized AD-related genes (*CD3EAP*, *TOMM40* and *SNRPD2*) and module 3 (M3) contained 1/24 prioritized AD-related genes (*KAT8*) (Fig 5C). In the enrichment analysis (*q*_*c*_ < 0.05, BH-FDR corrected), M1 genes were enriched for neurogenesis-, neuron differentiation-, amyloid-beta formation- and dephosphorylation-related processes, suggesting that a large proportion of detected AD-related genes aggregated in the neuron-relevant functional module and critical processes for AD in the hippocampus. M2 genes were enriched for ribonucleoprotein complex- and protein localization to mitochondrion-related processes. M3 genes were enriched for autophagy-, immune system development- and histone modification-related processes (S4 Table), indicating that several detected AD-related genes could modulate common cellular functions in the hippocampus.

### Hippocampal gene expression differences in ADNI data

In the TWAS and FOCUS analyses, we prioritized 24 genes with significant differences in the predicted *cis*-GReX in the hippocampus between the AD and control groups. We further validated this finding in ADNI imaging genetics dataset (http://www.loni.usc.edu/). We used the genotyping data, structural brain MRI data and demographic information from ADNI1, ADNIGO and ADNI2. After quality control and preprocessing of genetic and hippocampal volume data from brain MRI (see Materials and methods), 1410 ADNI subjects were finally included. At baseline, the 1410 ADNI subjects were diagnosed as cognitively normal (CN, n = 415), mild cognitive impairment (MCI, n = 720) and AD (n = 275). After up to 13 years of follow-up, the diagnoses were 317 CN, 416 MCI and 599 AD, and 78 individuals were excluded due to uncertain diagnoses. The baseline MCI patients (n = 567) with a follow-up period of more than 2 years were further divided into the conversion (MCI-C, n = 300) group and the stable (MCI-S, n = 267) group. The demographic information of the 1332 subjects with definite diagnoses is shown in Table 1.

**Table 1:** Demographics and MRI data of the included sample.

Demographic variables	CN	MCI	AD
Number	317	416	599
Age (years)	74.18 ± 5.63	73.75 ± 7.40	74.64 ± 7.35
Sex (Male/Female)	157/160	256/160	354/245
Education (years)	16.50 ± 2.68	16.01 ± 2.90	15.53 ± 2.88
Hippocampal volume (ml)	3738.88 ± 445.46	3474.60 ± 529.06	3002.31 ± 538.27

Data are shown as mean ± standard deviation.

CN = cognitively normal, MCI = mild cognitive impairment, AD = Alzheimer’s disease.

**Fig 6:**
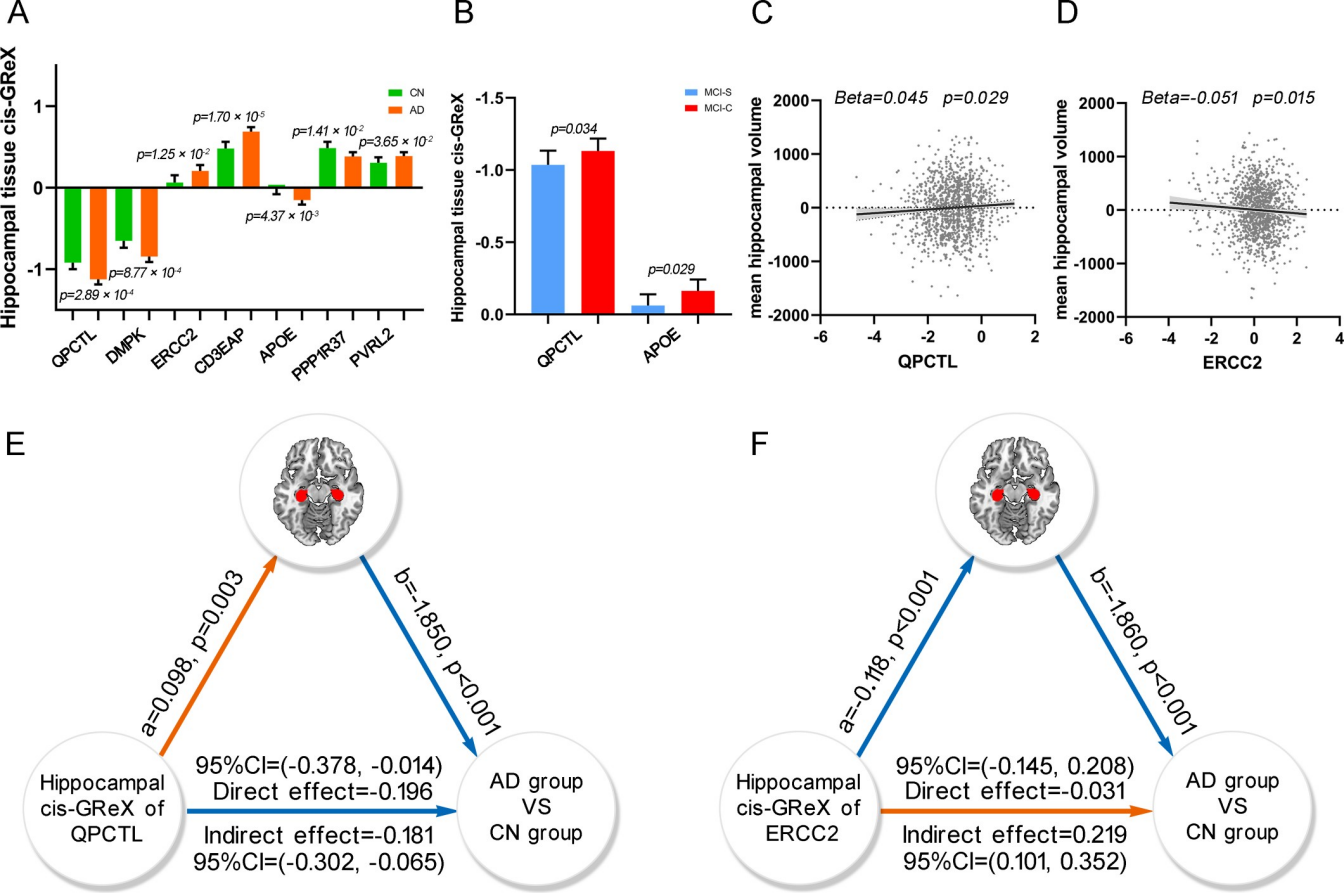
ADNI neuroimaging data analysis. (A) The bar plot shows the significant difference of hippocampal tissue *cis*-GReX of *QPCTL*, *DMPK*, *ERCC2*, *CD3EAP*, *APOE*, *PPP1R37* and *PVRL2* between AD and CN groups. The *p*-values are calculated by binary logistic regression between the hippocampal tissue *cis*-GReX of the seven genes and diagnoses. (B) The bar plot shows the significant difference of hippocampal tissue *cis*-GReX of *QPCTL* and *APOE* between MCI-C and MCI-S groups. (C and D) The scatter plots show correlations between the hippocampal tissue *cis*-GReX of *QPCTL* (C), *ERCC2* (D) and mean hippocampal volume using linear regression. The y-axis shows the residual of mean hippocampal volume after regressed age, sex, education, MR field strength, first 4 components of MDS and clinical diagnoses. (E and F) The mediation analysis shows that hippocampal volume mediates the effect of the hippocampal tissue *cis*-GReX of *QPCTL* (E) and *ERCC2* (F) on the diagnosis of AD. The colors of the lines demonstrate the positive correlation (orange color) and the negative correlation (blue color) in the analysis. AD, Alzheimer’s disease; *cis*-GReX, *cis*-genetically regulated expression; CN, cognitively normal; MCI-C, mild cognitive impairment conversion; MCI-S, mild cognitive impairment stable; MDS, multidimensional scaling.

For each gene, Predixcan [14] was used to predict the *cis*-GReX of the gene in hippocampal tissue for each ADNI subject by integrating genotypic data of the subject with the weighted value of each SNP derived from the prediction models. For each of the 24 AD-related genes, binary logistic regression was performed to test the difference in gene expression in hippocampal tissue (predicted *cis*-GReX) between the AD (n = 599) and CN (n = 317) groups, controlling for age, sex, education and the first 4 components of multidimensional scaling (MDS). The hippocampal expression of *QPCTL*, *DMPK*, *ERCC2*, *CD3EAP*, *APOE*, *PPP1R37* and *PVRL2* was significantly different between the two groups (Table 2 and Fig 6A). The hippocampal expression of *QPCTL* and *APOE* was also significantly different between the MCI-C and MCI-S groups (Fig 6B).

**Table 2:** Seven TWAS significant genes were validated in the ADNI neuroimaging data.

Gene	AD TWAS		ADNI validation			
	TWAS_Z	TWAS_P	OR	95%CI	SE	P
*QPCTL*	-23.332	2.10 × 10^−120^	0.760	0.656 to 0.882	0.076	2.89 × 10^−4^
*DMPK*	-22.672	8.54 × 10^−114^	0.774	0.666 to 0.900	0.077	8.77 × 10^−4^
*ERCC2*	17.870	2.01× 10^−71^	1.199	1.040 to 1.382	0.073	1.25 × 10^−2^
*CD3EAP*	14.916	2.60× 10^−50^	1.396	1.199 to 1.625	0.078	1.70 × 10^−5^
*APOE*	-12.901	4.43× 10^−38^	0.814	0.707 to 0.938	0.072	4.37 × 10^−3^
*PPP1R37*	-6.710	1.95× 10^−11^	0.834	0.722 to 0.964	0.074	1.41 × 10^−2^
*PVRL2*	4.458	8.26× 10^−6^	1.166	1.010 to 1.346	0.073	3.65 × 10^−2^

OR-values, 95% CI, SE-values and P-values are from binary logistic regression.

AD = Alzheimer’s disease, ADNI = Alzheimer’s Disease Neuroimaging Initiative

TWAS = transcriptome-wide association study, OR = odds ratio, CI = confidence interval, SE = standard error.

The abbreviation of genes is referred to at https://www.ncbi.nlm.nih.gov/gen.

### Hippocampal gene expression and hippocampal volume

Since the identified AD-related genes showed abnormal expression in the hippocampus and AD is characterized by hippocampal atrophy, we further wanted to identify which of these genes are associated with hippocampal volume. Seven genes were validated in ADNI whose expression in hippocampal tissue was associated with AD. We performed linear regression between the expression of each validated AD-related gene in the hippocampus and the mean hippocampal volume in the 1332 ADNI subjects while controlling for age, sex, education, MR field strength, clinical diagnosis and the first 4 components of MDS. We found that the mean hippocampal volume was nominally correlated with the expression of *QPCTL* (Beta = 0.045, *p* = 0.029) and *ERCC2* (Beta = -0.051, *p* = 0.015) in the hippocampus (Fig 6C and 6D). In addition, we performed multiple linear regression to identify the total effect of the 24 genes on the mean hippocampal volume of AD (n = 599), controlling for all confounders. The 24 genes impacted the hippocampal volume of AD (*p* = 0.039, R^2^ = 0.062). R^2^ is the proportion of variance of the dependent variable (hippocampal volume) that can be explained by the independent variables (predicted *cis*-GReX in hippocampal tissue of the 24 genes).

Given that the expression of *QPCTL* and *ERCC2* in hippocampal tissue was associated with hippocampal volume, we further explored whether the expression of these two genes in other subcortical tissues was also associated with the volumes of the corresponding structures. We used the same procedure as that of the hippocampus, wherein the predicted *cis*-GReX for each gene was calculated by integrating genotypic data of the ADNI subjects and the prediction models of the amygdala, caudate, nucleus accumbens and putamen, respectively. For the 1332 subjects we included, two individuals were excluded due to the failure of the segmentation of the four brain structures. We explored the correlations between the predicted *cis*-GReX of the two genes (*QPCTL*, *ERCC2*) in each of the three brain tissues (caudate, nucleus accumbens and putamen) and the corresponding volumes (n = 1330). There was no significant correlation between the *cis*-GReX of the two genes in these tissues and corresponding brain structure volumes (S5 Table). In contrast to the significant joint effect of the 24 prioritized AD-related genes on the hippocampal volume of AD, these genes showed no joint effect on the volumes of the amygdala, caudate, nucleus accumbens and putamen in AD patients (n = 599) (S7 Fig). Taken together, these results suggest that these genes show greater impacts on hippocampal volume than volumes of other subcortical nuclei.

### Hippocampal volume mediates the effect of hippocampal gene expression on AD

As an important intermediate phenotype of AD, hippocampal volume may mediate the association between gene expression (*QPCTL* and *ERCC2*) in hippocampal tissue and AD. To identify the hippocampus-mediated pathway from gene expression to AD, we performed mediation analysis, in which the predicted *cis*-GReX of *QPCTL* or *ERCC2* in hippocampal tissue was set as an independent variable, mean hippocampal volume as a mediator variable, and disease state (AD versus CN) as a dependent variable. The significance of the indirect effect was tested by calculating bias-corrected 95% bootstrap confidence interval with 5000 resampling, and the statistical significance of other effects was set at *p* < 0.05. For *QPCTL*, we found a significant indirect effect (effect = -0.181, 95% CI = -0.302 to -0.065) from *cis*-GReX to the disease state, indicating that the expression of *QPCTL* in hippocampal tissue could affect AD via modulating hippocampal volume. In addition, the direct effect (effect = -0.196, *p* = 0.034, 95% CI = -0.378 to -0.014) from the *cis*-GReX of *QPCTL* in hippocampus to the disease state was also significant, which represented a portion of the effect of the gene expression on AD being not mediated by hippocampal volume (Fig 6E). For *ERCC2*, the hippocampal volume could mediate the effect of the *cis*-GReX on the disease state with a significant indirect effect (effect = 0.219, 95% CI = 0.101 to 0.352), but the direct effect from the *cis*-GReX of *ERCC2* on the disease state was not significant (*p* = 0.727, 95% CI = -0.145 to 0.208) (Fig 6F). These findings indicate that the expression of *ERCC2* in hippocampal tissue could affect AD mainly by modulating hippocampal volume.

## Discussion

In this study, we jointly used TWAS and fine mapping approaches to identify genes whose expression in hippocampal tissue was associated with AD and screened 24 AD- and hippocampus-related genes involved in crucial biological processes of AD and functional modules in hippocampal tissue. We further validated the associations of *QPCTL*, *DMPK*, *ERCC2*, *CD3EAP*, *APOE*, *PPP1R37* and *PVRL2* with AD in ADNI data, and found relations of *QPCTL* and *APOE* with the conversion from MCI to AD. We also found that hippocampal volume mediated the associations of hippocampal tissue *cis*-GReX of *ERCC2* and *QPCTL* with AD. These findings provide candidate genes linked to AD by regulating gene expression in hippocampal tissue and underline the importance of the hippocampus in explaining the genetic risks of AD.

This study extends AD-related genetic loci identified by prior AD-GWAS studies [4–6,8–11] by providing evidence that some loci (*APOE*, *TOMM40*, *PVRL2*, *EXOC3L2*, *KAT8* and *HLA-DRB5*) may lead to AD by affecting the gene expression levels in the hippocampus. Among the 24 AD- and hippocampus-related genes identified in this study, previous studies only provide clues for the associations of hippocampal expression of *PRSS36*, *KAT8*, *HLA-DRB5* [4], *TOMM40* [20], *CEACAM19* and *PVRL2* [21] with AD. However, some indirect evidence may support other associations. For example, we found that the expression of *APOE* in hippocampal tissue was associated with AD and the conversion from MCI to AD, which is consistent with the concept that *APOE* is an important genetic risk gene for AD [36] and with the reduced APOE protein level in the hippocampus in patients with AD [37]. In addition, we identified 18/24 genes that have not been found in GWAS, which may be due to differences in methodology or the lack of statistical power in GWAS. However, our findings are highly consistent (19/24) with previous TWAS results [17–21].

Comparing with previous TWAS and GWAS studies, we found two novel genes, *PTPN9* and *PCDHA4*, affecting AD through hippocampal expression. Given the two loci are non-significant in AD GWASs, our analysis leveraged the hippocampal gene expression data and combined the effects of SNPs on each gene by TWAS to increase statistical power for discovery. Our prediction models successfully established the relationship between SNPs and gene expression for *PTPN9* and *PCDHA4*. In addition, compared to the other four brain tissues, the expression of the two genes was associated with AD only in hippocampal tissue, suggesting that mechanistically related tissue and high-performance prediction models of TWAS are important for identifying context-specific disease genes. The functional annotation revealed that *PTPN9* participated in neurogenesis (GO:0022008) and dephosphorylation (GO:0016311), and both *PTPN9* and *PCDHA4* were involved in nervous system development (GO:0007399). Modulization analysis based on the extended AD-related gene set and hippocampal-based network revealed that *PTPN9* was a member of the M1 functional module affecting neuron-related biological processes. *PTPN9* belongs to the protein tyrosine phosphatase family, which is involved in numerous important biological processes [38], and *PTPN9* knockout mice show severe neurodevelopmental disorders [39]. In addition, *PCDHA4* is a member of the protocadherin alpha gene family, and neural cadherin-like cell adhesion proteins encoded by these genes play a critical role in establishing complex brain networks of neuronal connections [40]. Knockdown of mouse protocadherin alpha proteins results in abnormalities in learning and memory [41]. Together, both functional annotation and previous studies provided evidence that *PTPN9* and *PCDHA4* may affect hippocampus-dependent AD development.

By combining network topology-based analysis, statistical over-representation test and hippocampal-based functional module detection, we can better understand the function of the identified AD- and hippocampus-related genes. These genes were interconnected in the PPI network and interacted with the causal proteins of AD, such as a key PPI network member, APP, which could generate neurotoxic Aβ peptide and play a crucial role in the development of AD [33,42]. The component genes of the constructed PPI network were related to many important processes for AD, such as amyloid-beta formation- and phosphorylation/dephosphorylation-related biological processes. In the AD brain, phosphorylation/dephosphorylation imbalance is an important mechanism for hyperphosphorylation of tau [43]. In addition, neuronal apoptosis- and neurogenesis-related processes have been identified, and in the AD brain, adult hippocampal neurogenesis is impaired with immature differentiation of neurons [44]. Telomerase is expressed in mature human hippocampal neurons [45], and telomerase-deficient mice with short telomeres exhibit loss of neurons in the hippocampus [46]. Neuronal telomeres are shorter in hippocampal neurons of AD [47]. Therefore, telomere-related processes may participate in AD pathogenesis. Moreover, 17/24 prioritized AD-related genes were involved in hippocampal-based functional modules and enriched in key pathways of AD, further supporting their pathogenicity in the etiology of AD.

In the present study, we also investigated the relationship of the predicted gene expression in hippocampal tissue with hippocampal volume in ADNI data. We found that *QPCTL* and *ERCC2* were associated with hippocampal volume and that hippocampal volume mediated the effect of the two genes on AD. The 24 AD- and hippocampus-related genes had combined effects on the hippocampal volume of AD. These associations were only found in the hippocampus (compared with the amygdala, caudate, nucleus accumbens and putamen). Thus, hippocampal volume, which is an important endophenotype of AD, could fill gaps between gene expression in hippocampal tissue and AD. In our analysis, *QPCTL* interacted with *APP*, and *QPCTL* and *ERCC2* were involved in M1, which is related to many important pathways for AD. Thus, the two genes may affect hippocampal volume by modulating neurogenesis, neuron differentiation, amyloid-beta formation and dephosphorylation related processes and further increase the risk of AD.

There are limitations in our study. First, although probabilistic fine-mapping was used in this study, it only yields credible sets of genes that contain potential causal genes by estimating the probability of causality, so it could be used to prioritize genes rather than to identify true causal genes. Further biological validation of the discovered genes needs to be performed in future studies. Second, the discovery and replication samples are partially overlapped (11.9% of the discovery sample and 84.7% of the replication sample). The discovery patients contain the AD-by-proxy phenotype, and the replication patients have defined diagnosis. We used the GWAS summary data with the largest sample size as the discovery sample to increase the statistical power. Due to the sample overlap, the replication sample could exclude the influence of AD-by-proxy phenotype, but not replicate the results in an independent dataset, therefore, the reproducibility of the identified genes in different dataset is challenged. To eliminate the effect of sample overlap on the reliability of the TWAS results, we used two independent databases to validate our results and successfully replicated 20/36 TWAS genes (S1 Text and S6 Table). In addition, we used the multiple trait analysis of GWAS (MTAG) [48] approach to perform meta-analysis using discovery and replication GWAS summary statistics while accounting for potential sample overlap, and 25/36 genes were successfully replicated (*q*_*c*_ < 0.05) (S1 Text and S7 Table). Taken together, we could replicate 31/36 of our identified genes (S8–S10 Figs); however, a completely independent large-scale GWAS data of AD will be needed to fully validate our discovery.

## Materials and methods

### Ethics statement

All the data used in this study were obtained from public data repositories and got approval by their medical ethics review committees. Details about informed consent of GTEx can be found in the original paper [22] (dbGaP accession number phs000424.v7. p2). For ADNI, written informed consent was provided for all participants, and the study protocol was approved by each participating sites’ institutional review board (http://www.loni.usc.edu/). For the GWAS summary data of AD, all cohorts obtained written informed consent and each study protocol was approved by the institutional review boards. Full details can be found in the original paper [4,11]. We followed the instructions of accessing summary data on the websites (https://ctg.cncr.nl/software/summary_statistics, https://www.niagads.org/datasets/ng00075).

### Data resources

#### WGS and RNA-seq data of hippocampal tissue

WGS and RNA-seq data of 111 hippocampal samples from GTEx Version 7 were used to build prediction models for gene expression in hippocampal tissue based on genomic variants. The pipelines for processing WGS and RNA-seq data were available at the GTEx portal (https://gtexportal.org/home/). For WGS data, the reads were annotated according to the human reference genome (hg19/GRCh37). The sample-level quality control (QC) included genotyping call rate per individual (> 98%), sex concordance check and identity check. The SNP-level QC included SNP call rate (> 85%), Hardy-Weinberg equilibrium (HWE) (*p* > 1 × 10^−6^), minor allele frequency (MAF) (> 1%), and with non-ambiguous strand (no A/T or C/G SNPs). The obtained SNPs were pre-phased by SHAPEIT2 [49] and imputed by IMPUTE2 [50] with the 1000 Genomes Phase 3 reference panel. A total of 7,920,040 SNPs were finally selected from the imputed SNPs based on the criteria of biallelic and single-character allele codes only, non-ambiguous stranded SNPs, SNP call rate = 100%, HWE *p* > 1 × 10^−6^, MAF > 0.01 and IMPUTE2 info quality score > 0.8. GTEx standard quantification and QC procedures were conducted for hippocampal tissue RNA-seq data by GTEx consortium. All reads were aligned to the human reference genome (hg19/GRCh37) based on GENCODE v19 reference annotations [51] (https://www.gencodegenes.org/human/release_19.html). The same pipeline used for the hippocampus was applied to process WGS and RNA-seq data of the amygdala, caudate, putamen and nucleus accumbens (S8 Table).

#### GWAS summary data of AD

In the discovery stage, GWAS summary statistics of AD was derived from a meta-analysis collected from the PGC-ALZ (n = 17,477), IGAP (n = 54,162), ADSP (n = 7,506) and UKBB (n = 376,113), including 455,258 (71,880 AD or AD-by-proxy and 383,378 controls) unrelated individuals of European ancestry. Details about genotyping, quality control and genome-wide meta-analysis can be found in the original paper [4]. In UKBB, each proxy case had a clear family history of AD. In the validation stage, we replicated our findings in a GWAS meta-analysis of diagnosed AD from the updated IGAP (n = 63,926 including 21,982 AD and 41,944 controls) [11]. 54,162 participants from IGAP were overlapped between the discovery and validation samples.

#### Neuroimaging and genotyping data of ADNI

The Alzheimer’s Disease Neuroimaging Initiative (ADNI) is a comprehensive imaging genetics dataset containing genetic, neuroimaging, biochemical and clinical data. The genotyping data, structural brain MRI data and demographic information used in this study were downloaded from the ADNI data repository (http://www.loni.usc.edu/). ADNI was designed as an ongoing, longitudinal project. Initially, ADNI1 enrolled participants of CN, MCI and AD. Subsequent studies, including ADNIGO and ADNI2, further extended the study with additional cohorts and followed up with rollovers. The 757 participants from ADNI1 were genotyped by Illumina Human610-Quad BeadChip, and the 793 participants from ADNIGO/2 were genotyped by Illumina HumanOmniExpress BeadChip (http://www.illumina.com). The intensity data was processed with GenomeStudio v2009.1. Detailed information for the QC and processing procedures is shown in the S1 Text. After QC and imputation, 1423 individuals and 8,035,650 autosomal SNPs were retained for subsequent analysis.

The ADNI repository provides hippocampal volume data and volumetric data of the amygdala, caudate, nucleus accumbens and putamen calculated by FreeSurfer (http://surfer.nmr.mgh.harvard.edu/) with the pipeline for cross-sectional comparisons; for details, please see the manual (http://www.loni.usc.edu/). First, QC was performed for hippocampal volumetric data. One individual without hippocampal volume data, eleven individuals with failed segmentation and one individual without clinical data were excluded. In the remaining 1410 individuals, the hippocampal volume data of 1377 individuals were extracted from the baseline data, and those of 33 individuals were extracted from the nearest time point data to the baseline. Second, QC was performed for the volumetric data of the amygdala, caudate, nucleus accumbens and putamen in the 1410 individuals who had qualified hippocampal volumetric data, and two individuals were excluded due to the failure of brain tissue segmentation of these structures.

### Predicting hippocampal gene expression by SNPs

For each gene, conditional analysis in QTLtools (https://qtltools.github.io/qtltools/) [29] was used to identify *cis*-eQTLs with independent effects on gene expression in a *cis*-window of ± 1 Mb from the transcription start site (TSS). In this analysis, forward variable selection was used to decide the number of independent signals per gene expression at a moderate threshold (*p* < 0.01), and backward elimination was used to assign nearby variants to the independent signals. For each candidate SNP, the genotype of each sample was encoded as 0, 1 and 2 based on the counts of the effect allele. For the 111 hippocampal samples, we used the candidate SNPs to predict gene expression in hippocampal tissue with an additive genetic model. After *cis*-eQTLs mapping, the prediction models were only built for protein-coding genes (n = 15,831). Prediction models were built using the nested cross validated elastic-net procedure following the GTEx V7 pipeline (https://github.com/hakyimlab/PredictDB_Pipeline_GTEx_v7) [14]. First, the 10-fold cross-validated elastic-net was performed 5 times to estimate the significance of the models. The 111 hippocampal samples were split into 5 folds randomly, one-fold was removed at a time, the remaining samples (four folds) were used to train the prediction models by elastic-net with 10-fold cross-validation to tune the lambda parameter, and then the prediction models were applied to the samples of the removed fold to evaluate the correlations between the predicted and measured expression levels of the hippocampal samples. The performance of each prediction model was assessed by the average Pearson correlation coefficient between predicted and measured expression across subjects, which was the averaged value of the 5 times 10-fold nested cross validation tests. A prediction model was significant if the estimated p-value for the average Pearson correlation coefficient passed the multiple testing correction threshold of FWE (*p*_*c*_ < 0.05/15,831 = 3.16 × 10^−6^). In addition, the threshold value of the average Pearson correlation coefficient was greater than 0.1 to avoid the negative correlation according to the suggestion of the pipeline. Second, for each significant prediction model, a new elastic-net model was trained using 10-fold cross validation to tune the lambda parameter based on all hippocampal samples to calculate weights. The pipeline could avoid the bias caused by using the same data to tune the parameter and assess the performance. The same procedure as that used for hippocampal tissue was applied to establish prediction models for the tissues of amygdala (15,827 protein-coding genes), caudate (15,926 protein-coding genes), putamen (15,629 protein-coding genes) and nucleus accumbens (15,937 protein-coding genes). The sex, 15 expression residuals, top 3 genetic principal components and sequencing platforms were controlled during both eQTLs mapping and prediction model construction. The prediction models generated the weighted value of each candidate SNP’s relative contribution to the gene’s expression level in the corresponding tissue.

### Identifying AD-related genes by TWAS

In this study, TWAS was used to identify AD-related genes by testing correlations between *cis*-GReX and AD diagnosis with S-PrediXcan [15], which was embedded in the MetaXcan framework (https://github.com/hakyimlab/MetaXcan). In TWAS, SNP-AD associations were derived from GWAS summary data of AD, SNP-expression associations were assessed by the weighted value of each SNP to corresponding gene expression in the hippocampal and other tissues, and LD reference set was created by the prediction models. Multiple testing was corrected by FDR method (*q*_*c*_ < 0.05).

### Fine-mapping TWAS-identified AD-related genes

To exclude the possibility that TWAS-identified AD-related genes resulted from the genomic architecture of LD or co-regulation of gene models, FOCUS [30] was used to estimate the posterior inclusion probabilities for causality while accounting for the correlation structures of LD and co-regulation of gene expression prediction models. As a recommended strategy to improve the power, FOCUS also included SNP weights for gene expression in nonhippocampal tissues (PrediXcan weights of the GTEx v7 data (http://predictdb.org/) [14] and FUSION (functional summary-based imputation) weights of the METSIM, NTR, YFS, CMC data (http://gusevlab.org/projects/fusion/) [16]. FOCUS estimated credible gene sets based on the posterior inclusion probabilities at the 90% confidence level.

### Network topology-based analysis

We conducted network topology-based analysis using Webgestalt [32] with reference to the human PPI of the Biological General Repository for Interaction Datasets (BIOGRID) (Build 3.5.167) [52]. For the seed genes we mapped to the PPI network, random walk analysis was performed to expand the network by ranking all genes based on their network proximity with the seed genes. The resulting PPI network was constructed with the input seeds and the 50 top-ranking neighbors.

### Statistical over-representation test

The generated network was investigated by performing a statistical over-representation test using PANTHER classification system (v.14.0) [34] based on GO biological processes. For the genes in the PPI network, a statistical over-representation test was applied to detect statistical over representation of input genes compared to the human genome reference gene list. We used Fisher’s exact test to calculate the *p-*value based on the comparison between the number of input genes in a certain term and the number of reference genes in the same term. Fold enrichment represents the ratio of the value of observed gene number over that of expected. We used the BH-FDR correction for multiple testing (*q*_*c*_ < 0.05).

### Finding functional modules composed of the identified AD-related genes

Gene network analysis was used to test whether the identified AD-related genes were involved in certain cohesive gene clusters in hippocampal tissue by the HumanBase online tool [35]. Functional enrichment was performed for the resulting functional modules using GO terms. The statistical significance of each GO term was tested by one-sided Fisher’s exact test, and multiple testing was corrected by BH-FDR (*q*_*c*_ < 0.05).

### Mediation analysis

The PROCESS macro for SPSS (v3.4) was used for mediation analysis [53]. Only genes with both intergroup expression differences between the AD and CN groups (*p* < 0.05) and correlations with hippocampal volume (*p* < 0.05) were selected for the mediation analysis. In this model, the hippocampal *cis*-GReX for each gene was defined as an independent variable, the mean hippocampal volume as a mediator variable, the disease states (AD versus CN) as a binary dependent variable, hippocampal volume was adjusted by a linear regression with MR field strength, and the covariates included age, sex and education. For the dichotomous outcome in our analysis, PROCESS generated the direct effects, indirect effects, and paths from the mediator variables to the binary dependent variables by logistic regression. The coefficients between independent variables and mediator variables were estimated by ordinary least squares (OLS) regression.

Detailed protocols of the methods used above are available in https://dx.doi.org/10.17504/protocols.io.bp4amqse.

## Supporting information

S1 Table Results of TWAS and FOCUS. (XLSX)

S2 Table Genes in protein-protein interaction (PPI) network by network topology-based analysis. (XLSX)

S3 Table Enriched gene ontology (GO) terms of biological process by the statistical over-representation test of PANTHER. (XLSX)

S4 Table Five functional modules in the context of hippocampal tissue networks. (XLSX)

S5 Table Correlations between gene expression of *QPCTL* and *ERCC2* and volumes in four subcortical nuclei. (DOCX)

S6 Table TWAS results of two independent data sets of GWAS summary statistics of AD. (XLSX)

S7 Table TWAS results of the GWAS summary statistics from MTAG. (XLSX)

S8 Table Quality control and imputation of WGS data. (DOCX)

S9 Table The prediction model summary of hippocampus. (XLSX)

S10 Table The prediction model summary of amygdala. (XLSX)

S11 Table The prediction model summary of caudate. (XLSX)

S12 Table The prediction model summary of nucleus accumbens. (XLSX)

S13 Table The prediction model summary of putamen. (XLSX)

S14 Table TWAS summary statistics in discovery and validation stage. (XLSX)

S15 Table TWAS summary statistics of amygdala, caudate, nucleus accumbens and putamen. (XLSX)

S16 Table TWAS summary statistics of UKBB and meta-analysis of MTAG. (XLSX)

S1 Fig Manhattan plot of all TWAS associations in validation stage. Each point represents a single gene, with physical position in chromosome plotted on the x-axis and z-score of association statistics between gene and AD plotted on the y-axis. Significant associations (*p* < 0.05, FDR corrected) are labeled with gene names.
(PDF)

S2 Fig The average Pearson correlation coefficients between the predicted and observed expression levels of prediction models of the amygdala, caudate, nucleus accumbens and putamen. (PDF)

S3 Fig Manhattan plot of all TWAS associations based on amygdala tissue. (PDF)

S4 Fig Manhattan plot of all TWAS associations based on caudate tissue. (PDF)

S5 Fig Manhattan plot of all TWAS associations based on nucleus accumbens tissue. (PDF)

S6 Fig Manhattan plot of all TWAS associations based on putamen tissue. (PDF)

S7 Fig Multiple linear regression between the expression of the 24 genes and the volumes of hippocampus, amygdala, caudate, nucleus accumbens and putamen in AD patients. The x-axis shows the five subcortical tissues, the y-axis shows the R^2^ obtained from multiple linear regression, which represents the proportion of variance of the dependent variable that can be explained by the independent variables. R^2^, coefficient of determination.
(PDF)

S8 Fig The Venn diagram of the identified genes in the extended validation of TWAS results. The blue circle represents the 36 genes identified in the discovery stage of TWAS (*q*_*c*_ < 0.05, FDR corrected) and validated at nominal threshold of *p* < 0.05 with consistent direction of z-scores between discovery and validation stage; The grey circle represents the 25 genes identified by using two independent data sets of GWAS summary statistics of AD; The yellow circle represents the 74 genes identified by using the GWAS summary statistics accounting for sample overlap.
(PDF)

S9 Fig Manhattan plot of all TWAS associations using GWAS summary statistics of AD-by-proxy phenotype from UKBB and the hippocampal tissue prediction models. (PDF)

S10 Fig Manhattan plot of all TWAS associations using GWAS summary statistics from MTAG and the hippocampal tissue prediction models. (PDF)

S1 Text Quality control and imputation for genotype data from ADNI. Extended validation of TWAS results.
(DOCX)
