# Supplementary material for: Hippocampal transcriptome-wide association study and neurobiological pathway analysis for Alzheimer’s disease
Source: PLoS Genet. 2021 Feb 25;17(2):e1009363. doi: 10.1371/journal.pgen.1009363 (PMC7906391; doi:10.1371/journal.pgen.1009363)
Supplement: S5 Table — (DOCX) [file pgen.1009363.s005.docx]

**S5 Table. Correlations between gene expression of *QPCTL* and *ERCC2* and volumes in four subcortical nuclei.**

|  | *QPCTL* | | *ERCC2* | |
| --- | --- | --- | --- | --- |
|  | Beta | *P* | Beta | *P* |
| Caudate | 0.036 | 0.163 | -0.026 | 0.307 |
| Hippocampus | 0.045 | 0.029 | -0.051 | 0.015 |
| Nucleus accumbens | 0.024 | 0.301 | -0.004 | 0.868 |
| Putamen | -0.017 | 0.482 | -0.014 | 0.565 |

Beta-values and P-values are derived from linear regression.
