# Supplementary material for: Hippocampal transcriptome-wide association study and neurobiological pathway analysis for Alzheimer’s disease
Source: PLoS Genet. 2021 Feb 25;17(2):e1009363. doi: 10.1371/journal.pgen.1009363 (PMC7906391; doi:10.1371/journal.pgen.1009363)
Supplement: S8 Table — (DOCX) [file pgen.1009363.s008.docx]

**S8 Table. Quality control and imputation of WGS data.**

| Tissue | Sample size (Male/Female) | Samples passing sample-level QC | SNPs passing SNP-level QC | SNPs after imputation and QC |
| --- | --- | --- | --- | --- |
| Amygdala | 88 (57/31) | 88 | 9,646,162 | 8,578,041 |
| Caudate | 144 (101/43) | 144 | 9,849,672 | 8,667,599 |
| Accumbens | 130 (88/42) | 130 | 9,466,637 | 8,343,070 |
| Putamen | 111 (79/32) | 111 | 8,930,956 | 7,880,059 |

QC, Quality control; SNP, single nucleotide polymorphism.
