# Supplementary figures and images for: Hippocampal transcriptome-wide association study and neurobiological pathway analysis for Alzheimer’s disease
Source: PLoS Genet. 2021 Feb 25;17(2):e1009363. doi: 10.1371/journal.pgen.1009363 (PMC7906391; doi:10.1371/journal.pgen.1009363)

# AD TWAS IGAP

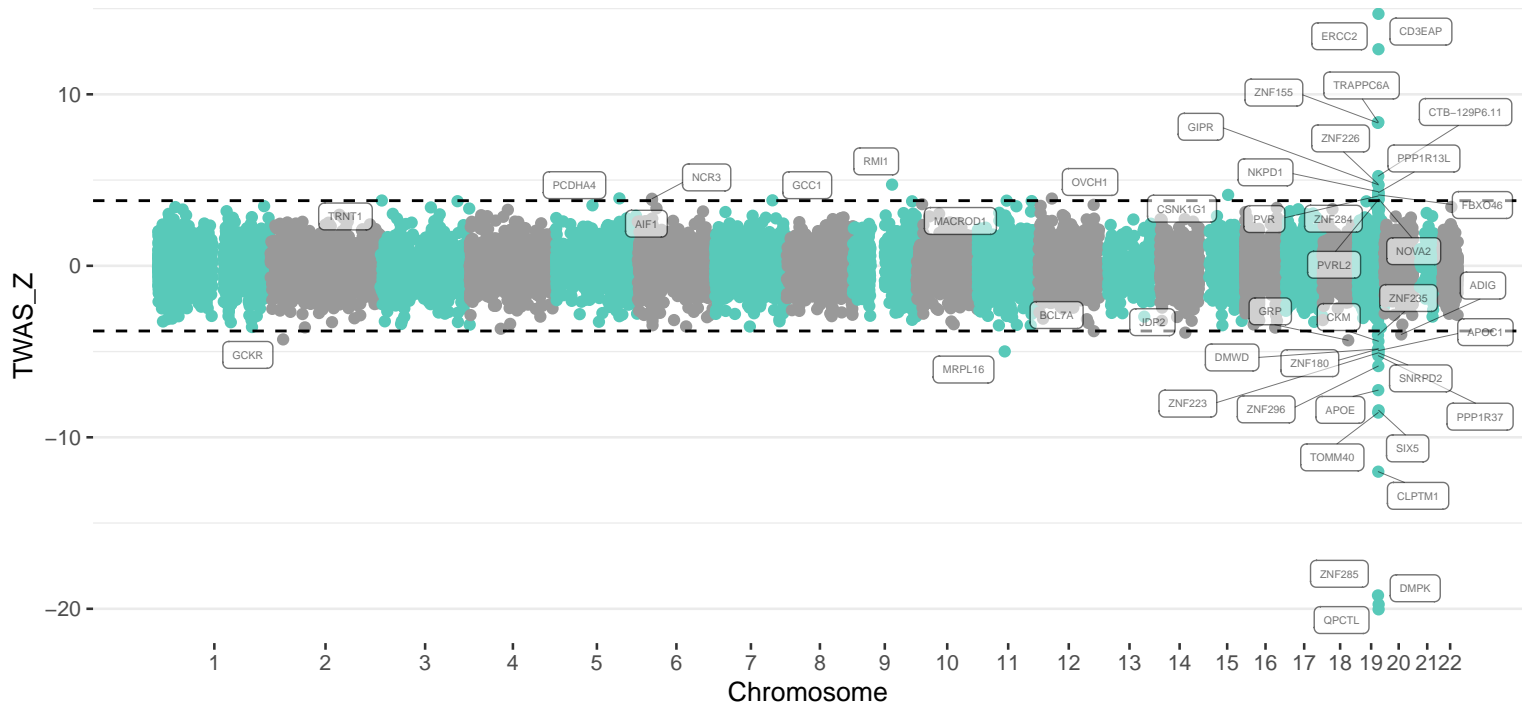

Supplement: S1 Fig — Each point represents a single gene, with physical position in chromosome plotted on the x-axis and z-score of association statistics between gene and AD plotted on the y-axis. Significant associations (p < 0.05, FDR corrected) are labeled with gene names. (PDF) [file pgen.1009363.s017.pdf]

A

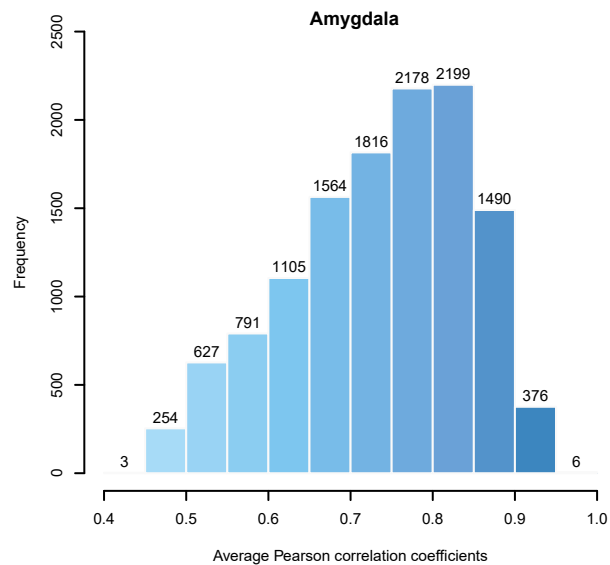

B

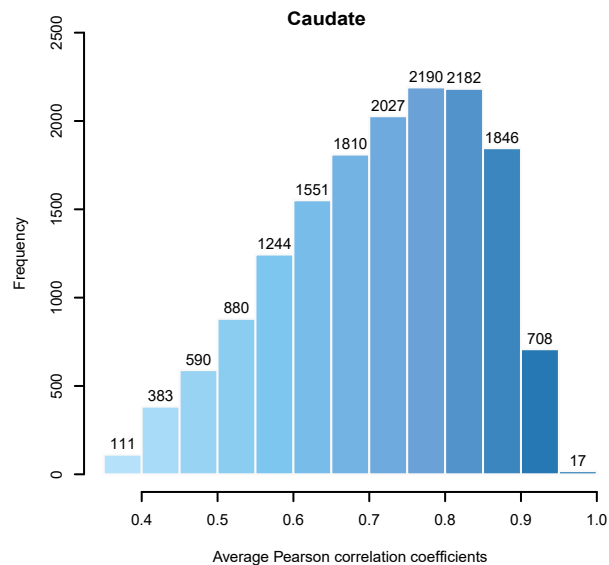

C

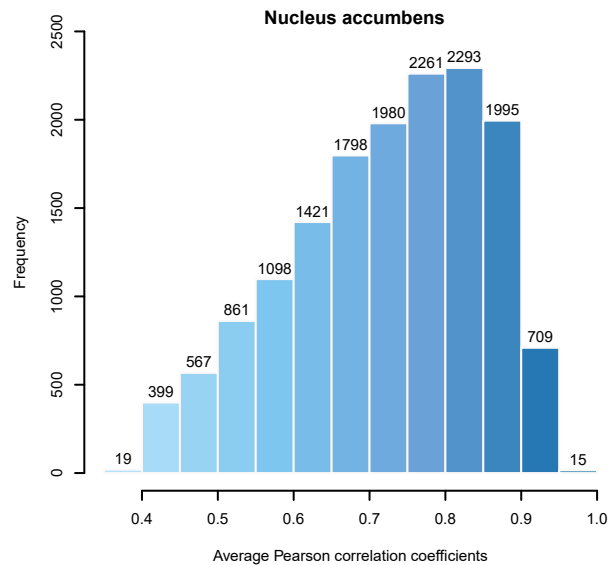

D

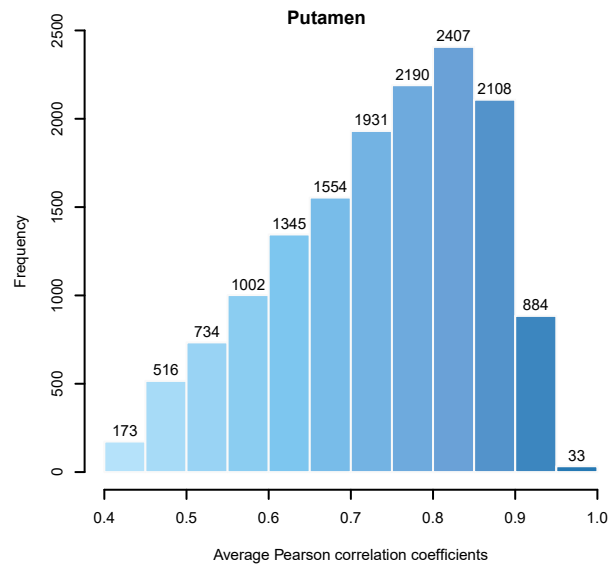

Supplement: S2 Fig — (PDF) [file pgen.1009363.s018.pdf]

# AD TWAS Amygdala

TWAS  $-\log_{10}(P)$

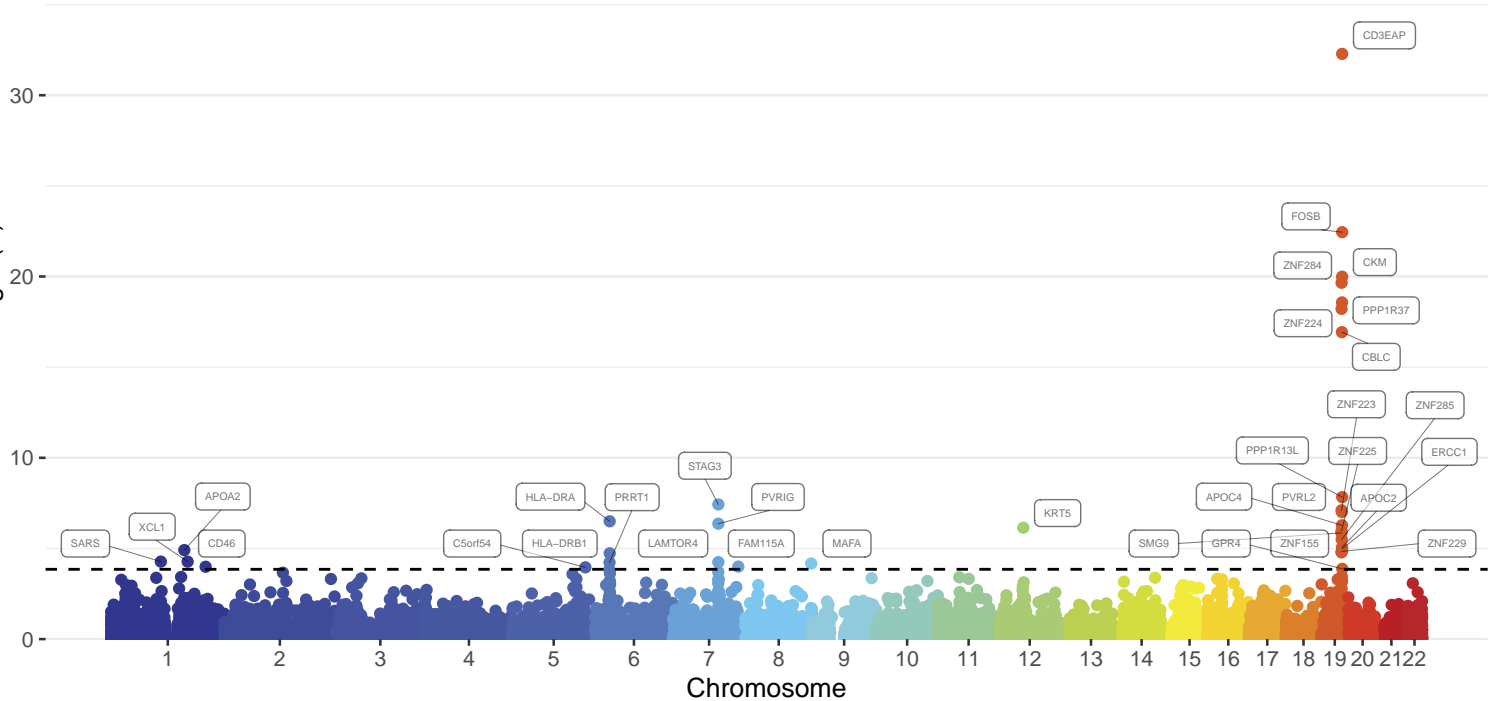

Supplement: S3 Fig — (PDF) [file pgen.1009363.s019.pdf]

# AD TWAS Caudate

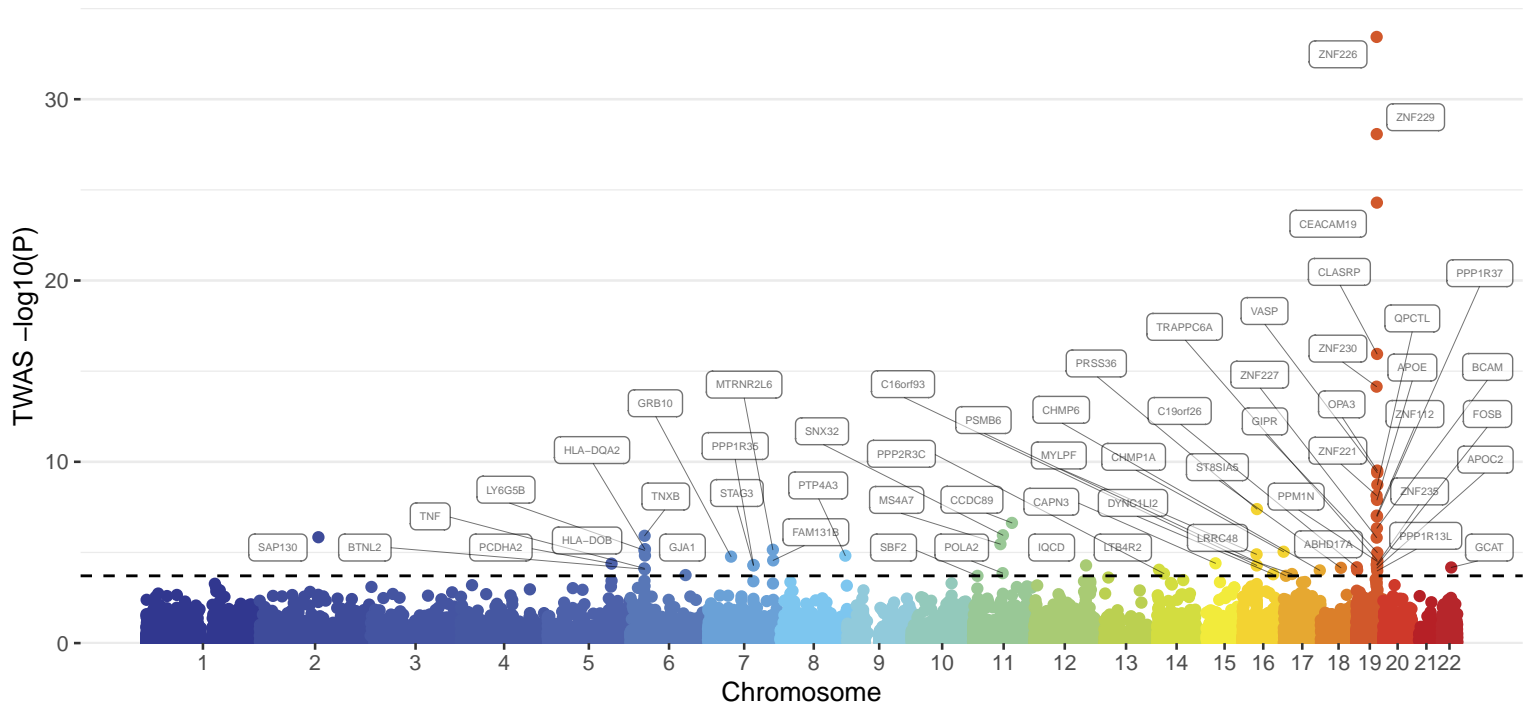

Supplement: S4 Fig — (PDF) [file pgen.1009363.s020.pdf]

# AD TWAS Nucleus accumbens

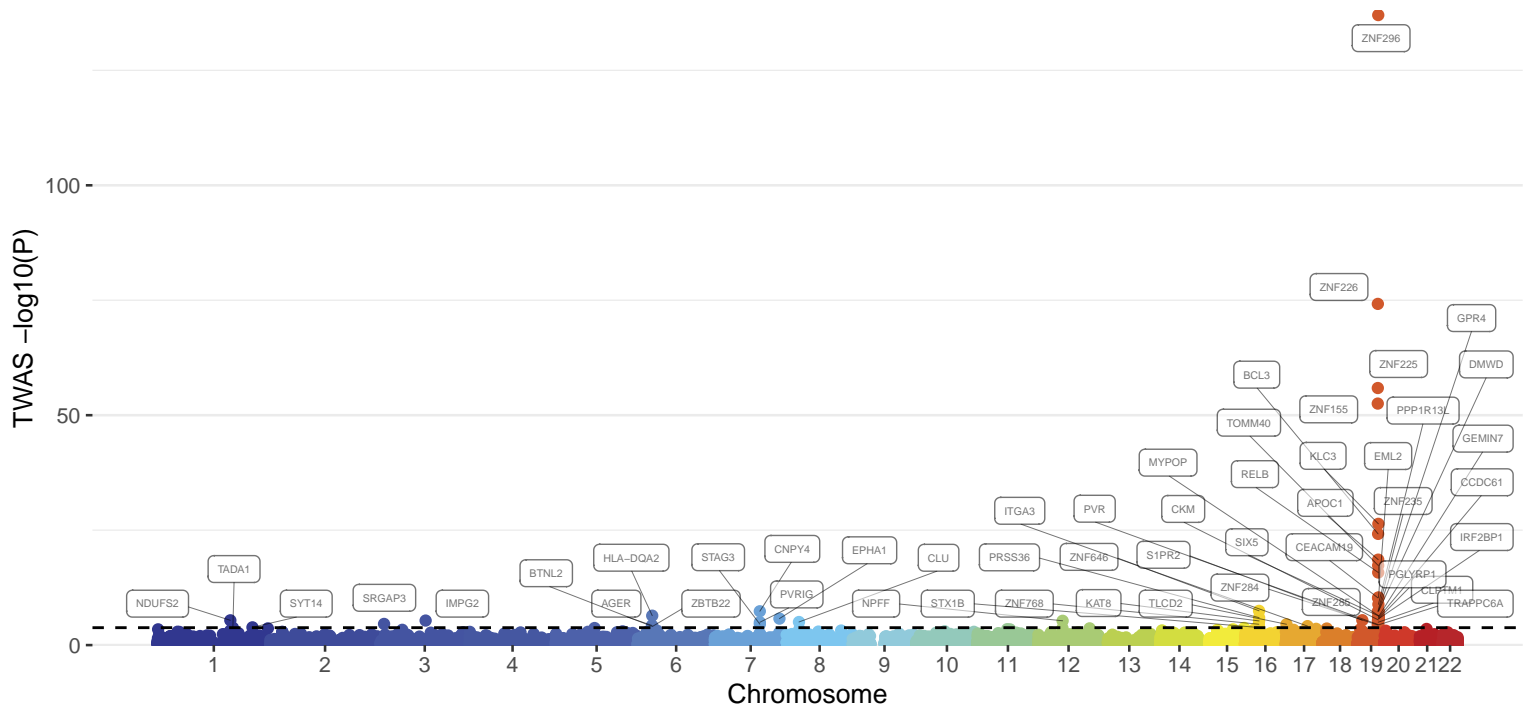

Supplement: S5 Fig — (PDF) [file pgen.1009363.s021.pdf]

# AD TWAS Putamen

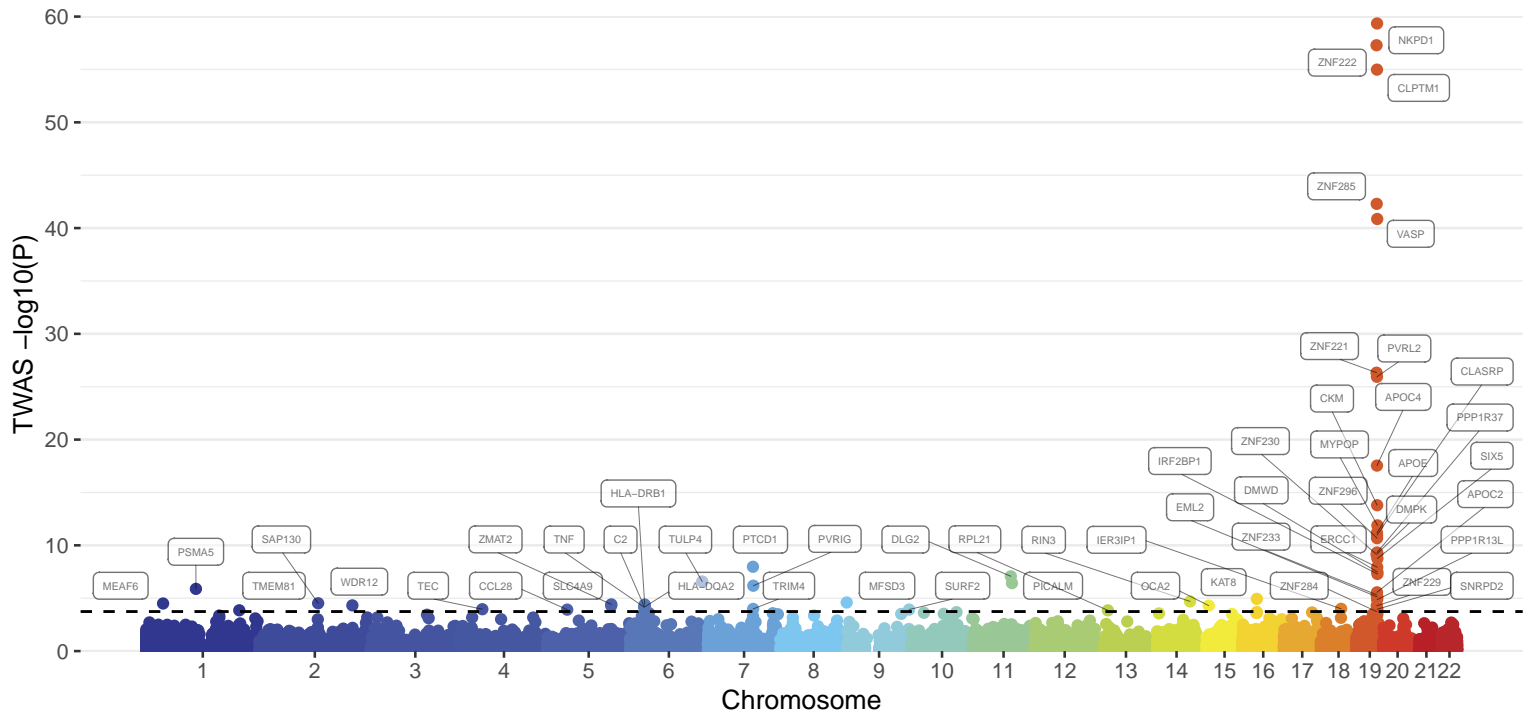

Supplement: S6 Fig — (PDF) [file pgen.1009363.s022.pdf]

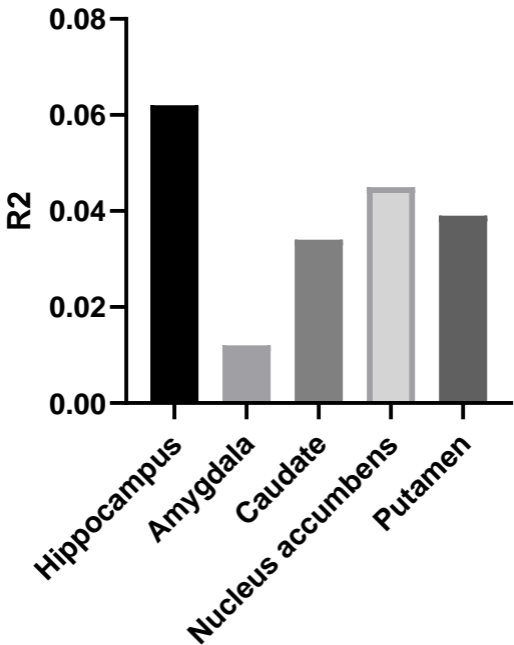

Supplement: S7 Fig — The x-axis shows the five subcortical tissues, the y-axis shows the R2 obtained from multiple linear regression, which represents the proportion of variance of the dependent variable that can be explained by the independent variables. R2, coefficient of determination. (PDF) [file pgen.1009363.s023.pdf]

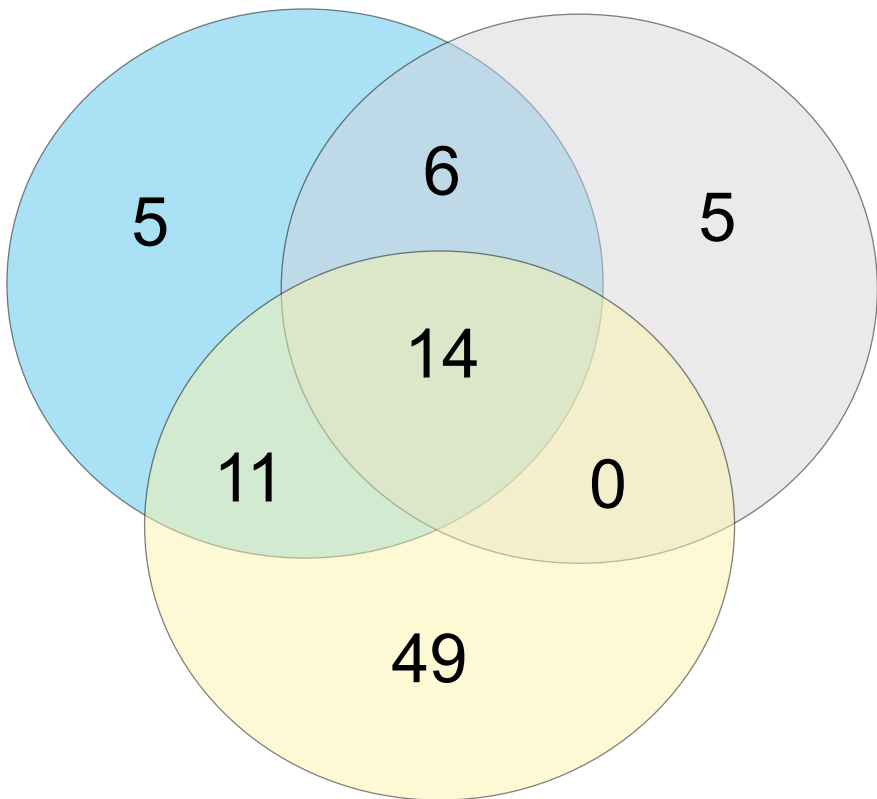

Supplement: S8 Fig — The blue circle represents the 36 genes identified in the discovery stage of TWAS (qc < 0.05, FDR corrected) and validated at nominal threshold of p < 0.05 with consistent direction of z-scores between discovery and validation stage; The grey circle represents the 25 genes identified by using two independent data sets of GWAS summary statistics of AD; The yellow circle represents the 74 genes identified by using the GWAS summary statistics accounting for sample overlap. (PDF) [file pgen.1009363.s024.pdf]

# AD TWAS UKBB

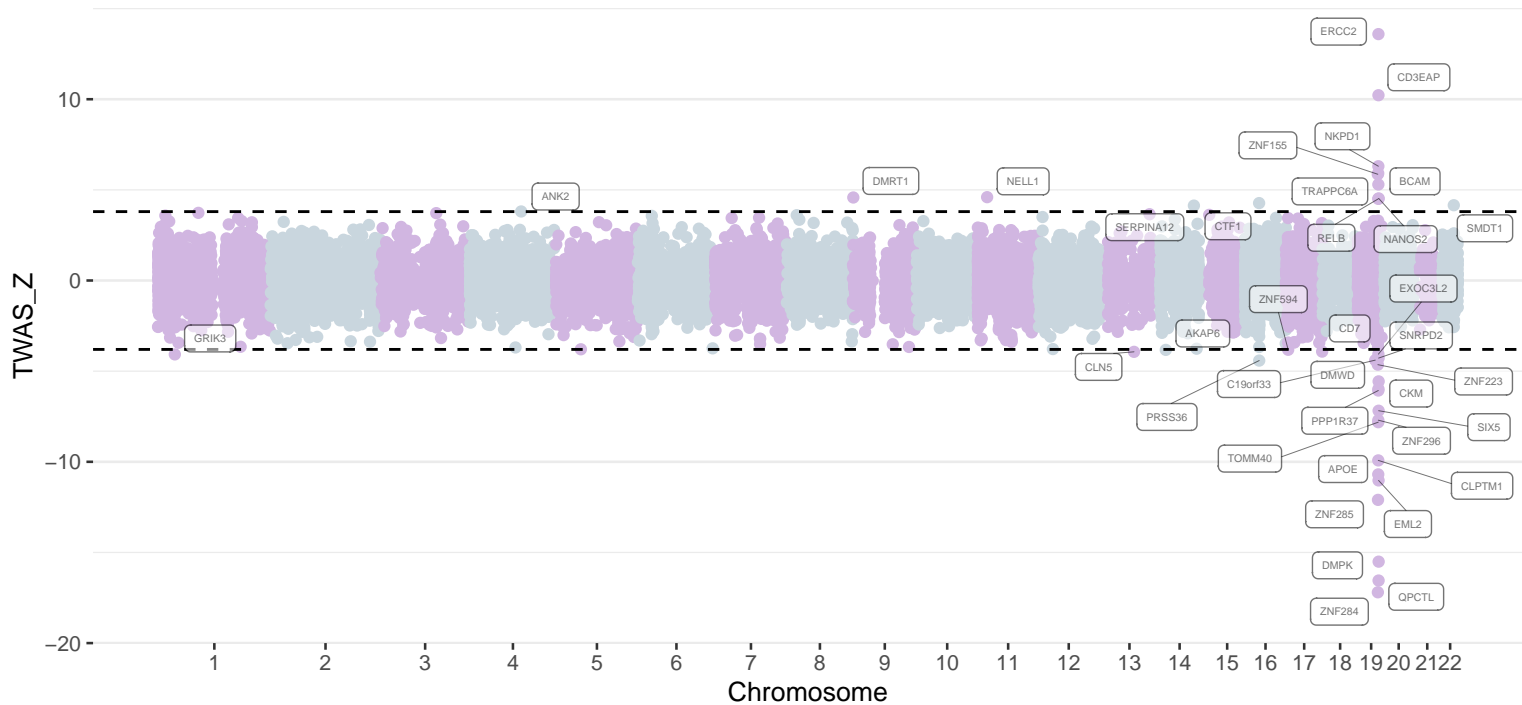

Supplement: S9 Fig — (PDF) [file pgen.1009363.s025.pdf]

# AD TWAS

TWAS  $-\log_{10}(P)$

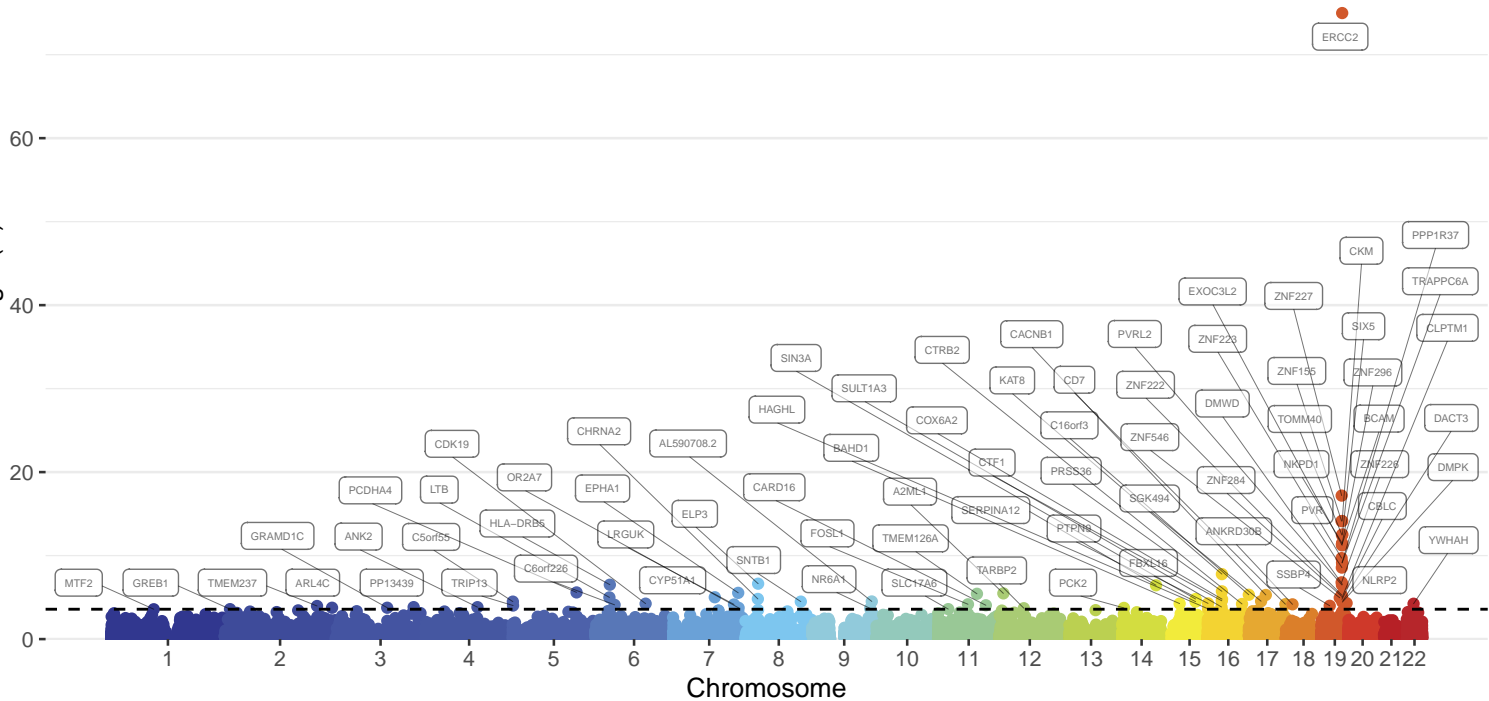

Supplement: S10 Fig — (PDF) [file pgen.1009363.s026.pdf]
