# Supplementary material for: Hippocampal transcriptome-wide association study and neurobiological pathway analysis for Alzheimer’s disease
Source: PLoS Genet. 2021 Feb 25;17(2):e1009363. doi: 10.1371/journal.pgen.1009363 (PMC7906391; doi:10.1371/journal.pgen.1009363)
Supplement: S1 Text — Extended validation of TWAS results. (DOCX) [file pgen.1009363.s027.docx]

**Hippocampal transcriptome-wide association study and neurobiological pathway analysis for** **Alzheimer's disease**

**Short title:** Hippocampal TWAS and neuroimage analysis for AD

Nana Liu^1¶^, Jiayuan Xu^1¶^, Huaigui Liu^1¶^, Shijie Zhang^2^, Miaoxin Li^3,4,5,6^, Yao Zhou^2^, Wen Qin^1^, Mulin Jun Li^2 *^ and Chunshui Yu^1,7 *^, for the Alzheimer's disease Neuroimaging Initiative^8^

1 Department of Radiology and Tianjin Key Laboratory of Functional Imaging, Tianjin Medical University General Hospital, Tianjin, China

2 The Province and Ministry Co-sponsored Collaborative Innovation Center for Medical Epigenetics, Tianjin Key Laboratory of Medical Epigenetics, Department of Pharmacology, Tianjin Medical University, Tianjin, China

3 Department of Medical Genetics, Center for Genome Research, Zhongshan School of Medicine, Sun Yat-sen University, Guangzhou, China

4 Centre for Genomic Sciences, The University of Hong Kong, Hong Kong Special Administrative Region, China

5 Department of Psychiatry, The University of Hong Kong, Hong Kong Special Administrative Region, China

6 Centre for Reproduction, Development and Growth, Li Ka Shing Faculty of Medicine, The University of Hong Kong, Hong Kong Special Administrative Region, China

7 Chinese Academy of Sciences (CAS) Center for Excellence in Brain Science and Intelligence Technology, Chinese Academy of Sciences, Shanghai, China

8 A complete list of Alzheimer's disease Neuroimaging Initiative investigators can be found at: <http://adni.loni.usc.edu/wp-content/uploads/how_to_apply/ADNI_Acknowledgement_List.pdf>

^¶^These authors contributed equally to this work.

***Correspondence to:**

* [chunshuiyu@tmu.edu.cn](mailto:chunshuiyu@tmu.edu.cn) (CY)

* [mulinli@connect.hku.hk](mailto:mulinli@connect.hku.hk) (MJL)

Content

1. Quality control and imputation for genotype data from ADNI

2. Extended validation of TWAS results

Quality control and imputation for genotype data from ADNI

Quality control for genotype data from ADNI1 and ADNIGO/2

For the genotype data from ADNI1, we used UCSC Genome Browser's liftover tool (<https://genome.ucsc.edu/cgi-bin/hgLiftOver>) [1] to map individual SNP markers to build 37 of the human reference genome，the following quality control was performed using the PLINK (version 1.90 beta6) (<https://www.cog-genomics.org/plink/>) [2]. The sample-level QC included genotyping call rate per individual (> 90%), sex concordance check and identity check by estimating of pairwise identity-by-descent (IBD). For the 757 subjects from ADNI1, eight individuals were excluded, two of them were excluded due to genetic correlation with individuals from ADNIGO/2. For the 793 subjects from ADNIGO/2, seven individuals were excluded. The SNP-level QC included SNP call rate (> 85%), Hardy-Weinberg equilibrium (HWE) (*p* > 1 × 10^-6^), minor allele frequency (MAF) (> 1%), and with non-ambiguous SNPs (no A/T or C/G). After sample- and SNP-level QC, A total of 749 (446 males) individuals and 550,834 variants (genotyping rate of 99.51%) in ADNI1 were included in the further imputation, and for ADNIGO/2, we retained 786 individuals (424 males) and 649,700 variants (genotyping rate of 99.83%) for following imputation.

Imputation and following QC

Pre-phasing was performed by SHAPEIT2 [3], imputation was performed by IMPUTE2 [4] with 1000 Genomes Phase 3 reference panel. We included SNPs with IMPUTE2 info quality score > 0.8, then merged ADNI1 and ADNIGO/2 genotype data. The merged genotype data was filtered with SNP call rate > 85%, HWE *p* > 1 × 10^-6^ and MAF > 0.01. We only included European population by assessing the population stratification through multidimensional scaling (MDS) analysis with HapMap phase III data (build 37 version) as the reference, 112 individuals were excluded, the first 4 components of MDS analysis were controlled for population stratification in the following analysis. Finally, 1423 individuals and 8,035,650 autosomal SNPs were retained for subsequent analysis.

Extended validation of TWAS results

To evaluate the effect of sample overlap of the discovery and replication GWAS summary data on the reliability of the results, we performed TWAS using two independent data sets of GWAS summary statistics of AD and the hippocampal tissue prediction models by summary-PrediXcan (S-PrediXcan) [5]. The GWAS summary statistics of IGAP (63,926 individuals including 21,982 AD and 41,944 controls) [6] was used as the discovery data. The GWAS summary statistics of AD-by-proxy phenotype, based on UKBB individuals for whom parental diagnostic status of AD were available (314,278 participants, 27,696 maternal cases, 14,338 paternal cases) [7], was used as the validation data. We found that the expression of 45 genes in hippocampal tissue were associated with AD (*q_c_* < 0.05, false discovery rate (FDR) corrected) (S1 Fig and S6 Table), among which 25 genes were replicated at a nominal threshold of *p* < 0.05 in UKBB data set and have consistent direction of z-scores between the two datasets (S9 Fig and S6 Table). Twenty of the newly obtained 25 genes were overlapped with the 36 genes we identified previously.

In addition, we performed meta-analysis using the multiple trait analysis of GWAS (MTAG) [8] approach, which can jointly analyze GWAS summary statistics with overlapping samples for the same trait or multiple correlated phenotypes. We used MTAG to combine the two GWAS summary statistics of AD (the meta-analysis of 455,258 participants and the IGAP of 63,926 participants), then the result of meta-analysis accounting for sample overlapping and the hippocampal tissue prediction models were used to perform TWAS. We found 31/36 genes associated with AD at a nominal threshold of *p* < 0.05, and 25/36 genes could pass the FDR correction (S10 Fig and S7 Table).

References

1. Hinrichs AS. The UCSC Genome Browser Database: update 2006. Nucleic Acids Research. 2006;34(90001):D590-D8. doi: 10.1093/nar/gkj144.

2. Purcell S, Neale B, Todd-Brown K, Thomas L, Ferreira MAR, Bender D, et al. PLINK: A Tool Set for Whole-Genome Association and Population-Based Linkage Analyses. The American Journal of Human Genetics. 2007;81(3):559-75. doi: 10.1086/519795.

3. Delaneau O, Zagury JF, Marchini J. Improved whole-chromosome phasing for disease and population genetic studies. Nat Methods. 2013;10(1):5-6. doi: 10.1038/nmeth.2307.

4. Howie BN, Donnelly P, Marchini J. A flexible and accurate genotype imputation method for the next generation of genome-wide association studies. PLoS Genet. 2009;5(6):e1000529. doi: 10.1371/journal.pgen.1000529.

5. Barbeira AN, Dickinson SP, Bonazzola R, Zheng J, Wheeler HE, Torres JM, et al. Exploring the phenotypic consequences of tissue specific gene expression variation inferred from GWAS summary statistics. Nat Commun. 2018;9(1):1825. doi: 10.1038/s41467-018-03621-1.

6. Kunkle BW, Grenier-Boley B, Sims R, Bis JC, Damotte V, Naj AC, et al. Genetic meta-analysis of diagnosed Alzheimer's disease identifies new risk loci and implicates Abeta, tau, immunity and lipid processing. Nat Genet. 2019;51(3):414-30. doi: 10.1038/s41588-019-0358-2.

7. Marioni RE, Harris SE, Zhang Q, McRae AF, Hagenaars SP, Hill WD, et al. GWAS on family history of Alzheimer's disease. Translational psychiatry. 2018;8(1):99. doi: 10.1038/s41398-018-0150-6.

8. Turley P, Walters RK, Maghzian O, Okbay A, Lee JJ, Fontana MA, et al. Multi-trait analysis of genome-wide association summary statistics using MTAG. Nat Genet. 2018;50(2):229-37. doi: 10.1038/s41588-017-0009-4.
